# Large variability of preincubation storage temperature in chukar partridge eggs under normal storage duration: Impact on egg and hatching traits, and embryonic development

**DOI:** 10.1371/journal.pone.0354754

**Published:** 2026-09-23

**Authors:** Mustafa Çam, Mehmet Özbek, Zahit Kutalmış Kaya, Harun Karaca, Mahmut Şamil Şamlı, Serdar Güler, Neffel Kürşat Akbulut, Kemal Kırıkçı

**Affiliations:** 1 Department of Animal Science, Faculty of Veterinary Medicine, Selcuk University, Konya, Turkiye; 2 Faculty of Food and Land Systems, Animal Welfare Program, University of British Columbia, Vancouver, Canada; 3 Department of Histology and Embryology, Faculty of Veterinary Medicine, Burdur Mehmet Akif Ersoy University, Burdur, Turkiye; 4 Department of Plant and Animal Production, Eregli Kemal Akman Vocational School Necmettin Erbakan University, Konya, Turkiye; 5 Department of Animal Science, Faculty of Veterinary Medicine, Mustafa Kemal University, Hatay, Turkiye; 6 Department of Animal Science, Faculty of Veterinary Medicine, Dokuz Eylül University, İzmir, Turkiye; 7 Department of Obstetrics and Gynaecology, Faculty of Veterinary Medicine, Necmettin Erbakan University, Konya, Turkiye; The University of Adelaide - North Terrace Campus: The University of Adelaide, AUSTRALIA

## Abstract

The effect of different storage temperatures on egg traits during storage and incubation, hatching characteristics and embryonic mortality in chukar partridge eggs stored under normal-term storage was investigated in this study. The study was designed as a 2 × 4 factorial arrangement with two storage durations (7 and 14 days) and four storage temperatures (3, 9, 15 and 21 °C). After storage, a total of 480 eggs (60 eggs per subgroup) were sent directly to the histology lab to determine the stage of embryonic development. The remaining eggs (50 eggs per each subgroup) were moved to an incubator for 21 d and then transferred to a hatcher basket. On day 26, unhatched eggs were opened to determine the hatching performance and embryonic mortality rate. Lower chick weights and longer incubation periods were observed when the eggs were stored at 15 °C. Almost all the egg and hatching traits were found to be similar among treatment groups. Considering that the probability of a higher embryonic developmental stage was increased in the eggs stored at 21 °C, the occurrence of embryonic development at gastrula stage was found to be similar across both storage duration and temperature groups. Even though storage temperature is recommended to be adjusted between 12–18 °C in poultry industry, to the best of our knowledge, this is the first study to claim that partridge eggs can be stored from 3 to 21 °C without any serious negative effect in the 14 days of storage period. Most insignificant findings in both hatchability and embryonic occurrence in eggs stored at 21 °C revealed that partridge breeders and game farmers would store their eggs during the short-term storage period by placing them in a room with minor temperature and humidity modifications.

## Introduction

In Mediterranean countries, partridges are mainly raised on game farms to provide stocks for hunting tourism and meat purposes as well [1,2]. The chukar partridge (*Alectoris chukar*) is a common gamebird in the family *Phasianidae* introduced in Eurasian countries [1]. Due to the limited seasonal laying period [3] and the high value of chicks [4], all partridge eggs should be incubated rather than marketed for sale. In addition, partridge egg production is low at the beginning and end of the laying season [5]. Therefore, preincubation storage of partridge eggs requires farmers to collect a sufficient number of eggs to load incubators and to raise the desired batch size of partridge chicks.

Several studies have been conducted to determine the optimum storage conditions for hatching partridge eggs. Partridge eggs have been reported to be more resistant to long storage periods than the other poultry species and can be stored at the temperatures ranging from 13 to 16 °C up to 21 days without any severe adverse effects on hatching results [6–12]. Moreover, Gomez-de-Travecedo et al [2] suggested that the storage temperature for partridge eggs should be lowered to 12 °C or lower under long-term storage conditions that is up to 42 d.

Physiological zero is defined as the temperature below which embryonic development ceases during storage and has been reported to be below 21 °C in poultry industry [13]. If the temperature is above physiological zero, abnormal embryonic development occurs, leading to arrested development because of early embryonic mortality. Embryonic development before incubation is defined as a staging method using a 14-level scoring system based on the morphological state of blastoderm development [14]. According to the same authors, embryonic development occurs when the eggs are in the gastrula stage of the development (EGK ≥ 10)

Physiological zero temperature may fluctuate at different temperatures during different storage periods. Özlü et al [15] reported that embryonic development advanced at a storage temperature of 18 °C when eggs were stored for long-term period.

There is a lack of information on both cold storage effects and preincubation embryonic development in partridge eggs. The objective of this study was to compare egg traits during incubation, hatching results and embryonic development of the partridge eggs stored at different temperatures (3, 9, 15 and 21 °C) under normal storage durations (7 and 14 days). We also aimed to test how partridge eggs would cease embryonic development at either the physiological zero temperature or below. Furthermore, egg traits obtained during incubation and hatching results would be linked to their relationships with embryonic development.

## Materials and methods

Ethical application for the study was approved by the Ethics Committee of Selcuk University Veterinary Faculty (SUVDAMEK, 2021/ 58) report.

### Partridge breeding and housing

The study was carried out at the Department of Poultry Breeding in Bahri Dagdas Agricultural Research Institute (37° 52’ 5.7612“ and 32° 33’ 12.8088”). The breeder partridges were raised in semi-open cages (10 males to 30 females/cage) with the dimension of 6.0 m long, 1.2 wide, and 1.2 height. The cages were located directly close to the experimental hatchery of the department. An artificial lightning program was created to stimulate natural mating after partridges reached 36 weeks of age. The artificial light per day was increased by 1 h per week until 16 h of light (natural + artificial) per day was achieved. All the partridges were reared under the same management conditions and feeding regime (Table 1) with ad-libitum access to feed and water during the entire breeding season. The eggs were collected twice daily.

**Table 1:** The ingredients and chemical composition of partridge diet.

Ingredients	%
Wheat	37.28
Maize	24.90
Boncalite	5.00
Vegetable oil	3.00
Soybean meal (%48)	19.10
Marble powder	6.37
Dicalcium phosphate 18	2.78
L-lysine hydrochloride	0.88
Salt	0.42
Vitamin-Mineral Premix^*^	0.25
DL-methionine	0.02
**Calculated nutrient concentration**	
ME (kcal/kg)	2800
CP (% KM)	18
Ca (%)	3.11
P (%)	0.61
Lysine (%)	1.5
Methionine + cystine (%)	0.6

* Premix provided the following per kg of diet: Vitamin A, 8.800 IU; vitamin D3, 2.200 IU; vitamin E, 11 mg; nicotinic acid, 44 mg; Cal-D Pantothenate, 8.8 mg; riboflavin, 4.4 mg; thiamine, 2.5 mg; vitamin B12, 6.6 mg; folic acid, 1 mg; D-Biotin, 0.11 mg; colin, 220 mg; Mn, 80 mg; Cu, 5 mg; Fe, 60 mg; Zn, 60 mg; Co, 0.20 mg; iodine, 1 mg; Se, 0.15 mg.

### Experimental design

The study was designed as a 2 × 4 factorial arrangement with 2 levels of storage durations (7 and 14 days) and 4 levels of storage temperatures (3, 9, 15 and 21 °C) treatments.

### Data collection during egg storage and incubation process

A total of 880 freshly laid eggs from 56-week-old chukar partridges were divided into two equal batches by collection at 7-day interval to form two storage duration groups at 14 and 7 days respectively. The eggs from each batch were numbered and weighed (0.01 g precision) individually and then randomly assigned to four storage chambers (HD-960L-3in1, Türkiye), each of which had different storage temperatures: 3, 9, 15 and 21 °C. Eggs weighing between 19 g and 24 g were included to the study [16].The eggs were stored with the position of pointed end up and turned 45° once daily at 75% relative humidity (RH) [8].

On the same day, all eggs from the two batches were taken out from the storage chambers and then weighed to obtain weight loss during storage. A random subset of 60 eggs from each subgroup was sent to the histology laboratory for the examination of embryo development. Partridge eggs were obtained and maintained under standardized environmental conditions until experimental procedures were initiated. After acceptance, the eggshells were opened delicately to avoid disruption, and the albumen and yolk were separated. The yolk was gently placed in a sterile petri dish containing pre-warmed Pannett-Compton saline to preserve tissue hydration and structural integrity. The blastoderm region was located via using a stereomicroscope. To minimize mechanical stress, the area encompassing the blastoderm was carefully lifted using a blotting paper. A square piece of filter paper was prepared and a central window was precisely cut to expose the blastoderm. The filter paper was gently positioned on the vitelline membrane to ensure that the blastoderm was centered. The filter paper, along with the vitelline membrane and the underlying blastoderm, was excised using microsurgical scissors. The isolated unit was transferred to a second Petri dish containing fresh Pannett-Compton saline. Any remaining yolk was carefully removed by gentle aspiration using a Pasteur pipette to provide a clear view of the membrane and embryonic disc. Initial morphological assessments were performed under a stereomicroscope (Leica S6D, Leica Microsystems GmbH, Wetzlar, Germany), while the sample remained attached to the filter paper. Subsequently, the vitelline membrane bearing the blastoderm was mounted onto poly-L-lysine-coated glass slides to enhance tissue adherence for microscopic analysis. Fixation was performed using 4% paraformaldehyde (PFA) prepared in phosphate-buffered saline (PBS) at room temperature. The samples were then briefly rinsed with PBS to remove residual fixative. Nuclear visualization was performed using DAPI staining, following the protocol outlined by Pokhrel et al [17]. Stained embryonic discs were examined under a fluorescence microscope (Olympus BX51) to evaluate nuclear organization and developmental progression. Developmental staging, from the early cleavage stages to primitive streak formation, was conducted based on the staging system proposed by Eyal-Giladi and Kochav [14], which provides a comprehensive reference for early avian embryogenesis. To ensure objectivity and reproducibility, all samples were independently evaluated and staged by two experienced histologists.

The remaining eggs were preheated for 12 h at room temperature and then loaded into a fumigator. The fumigation process was performed (formaldehyde + potassium permanganate) for 10 minutes and the eggs were set into an incubator (Çimuka T1600C, Türkiye). The eggs were incubated together at 37.7 °C and 59% RH with the position of blunt end up. The eggs were turned six times a day during incubation. After 21 days of incubation, each egg was weighed to estimate weight loss during incubation and put in a linen bag for exact identification during hatching. During both the storage and incubation processes, few cracked eggs were identified and excluded from the study. All the eggs were then transferred to the hatching baskets and then set into a hatcher (Çimuka T1600C, Türkiye). The eggs were hatched at 37.2 °C and 72% RH without any turning process. Hatched eggs were counted once a day up to day 26 to calculate the incubation length. All chicks were individually weighed to determine chick weight on their hatch day. On day 26, the unhatched eggs were counted and then broken for a rough macroscopic examination to determine unfertile and embryonic death [18]. The unfertile, embryonic mortalities according to each stage (early: 0–9^th^ days, middle: 9–21^st^ days; late: after 21 days, pip) were determined for the calculation of hatching results [2].

### Statistical analysis

The SPSS program (ver. 29.0) was used to perform statistical analyses. Egg traits during incubation were analysed by using Univariate GLM procedure with storage duration and temperature as fixed factors. Bonferroni post hoc test was applied for the comparisons between multiple groups. The developmental stage and occurrence of embryonic development were analysed by using Generalized Linear Model. The probability of having a higher developmental stage among the treatment groups was modelled by ordinal regression using a cumulative log link function and assuming a multinominal distribution. The occurrence of embryonic development (EGK > 10) was examined by binary logistic regression using a binominal distribution. Both the storage duration and temperature treatments and their interactions were added to all models as fixed effects. Hatching performances and embryonic deaths for each stage were analysed by using Pearson Chi Square test with Monte-Carlo method to obtain more accurate results. Statistical significances was set at P < 0.05 level with 95% confidence interval.

## Results

The descriptive statistics of the egg traits and the effects of different storage lengths and temperatures on chukar eggs are presented in Table 2. The eggs stored for 14 days lost more weight than those stored for 7 days (P < 0.001). As the storage temperature increased up to 15 °C, a gradual increase in the storage weight loss of eggs was observed (P < 0.001). Other parameters of egg traits showed no significant differences among the treatment groups. There was no significant interaction between length × temperature of storage for egg traits during incubation, except for egg weight loss during storage (P < 0.05).

**Table 2:** Changes in egg traits of chukar partridges during incubation under different storage temperature and duration.

SL (d)	ST (°C)	n	EWBS (g)	EWAS (g)	EW-I21 (g)	EWLS (%)	EWLI (%)	TEW (%)
**7**	**3**	50	21.45 ± 1.33	21.33 ± 1.33	18.77 ± 1.42	0.55 ± 0.15	12.07 ± 2.29	12.55 ± 2.38
	**9**	50	21.27 ± 1.27	21.11 ± 1.28	18.53 ± 1.87	0.78 ± 0.48	12.31 ± 6.11	12.96 ± 6.31
	**15**	48	20.95 ± 1.37	20.73 ± 1.37	18.17 ± 1.51	1.09 ± 0.36	12.39 ± 3.30	13.33 ± 3.52
	**21**	48	21.46 ± 1.07	21.24 ± 1.10	18.46 ± 1.63	1.04 ± 0.53	13.19 ± 4.89	14.06 ± 5.22
**14**	**3**	50	21.09 ± 1.18	20.89 ± 1.16	18.27 ± 1.09	0.93 ± 0.27	12.49 ± 2.70	13.30 ± 2.89
	**9**	49	21.23 ± 1.02	20.98 ± 1.02	18.37 ± 1.25	1.19 ± 0.37	12.45 ± 3.65	13.48 ± 3.88
	**15**	49	21.14 ± 1.32	20.77 ± 1.33	18.20 ± 1.42	1.72 ± 0.44	12.44 ± 2.45	13.93 ± 2.77
	**21**	48	21.13 ± 1.21	20.77 ± 1.23	18.27 ± 1.43	1.70 ± 0.37	12.12 ± 2.67	13.61 ± 2.92
**7**		196	21.28	21.10	18.48	0.86	12.49	13.23
**14**		196	21.14	20.85	18.28	1.39	12.37	13.58
**SEM**			0.09	0.09	0.11	0.03	0.27	0.40
**P-value**			–	*	–	***	–	–
	**3**	100	21.27	21.11	18.41	0,74^c^	12.28	13.05
	**9**	99	21.25	21.04	18.26	0,98^b^	12.38	13.59
	**15**	97	21.04	20.75	18.21	1.40^a^	12.41	13.53
	**21**	96	21.29	21.00	18.26	1.37^a^	12.65	13.60
**SEM**			0,12	0,13	0.15	0.04	0.38	0.40
**P-value**			–	–	–	***	–	–
**SL (d) × ST (°C)**			–	–	–	*	–	–

EWAS: Egg weight after storage, EWBS: Egg weight before storage, EW-I21: Egg weight at 21 days of incubation, EWLI: Egg weight loss during incubation, EWLS: Egg weight loss during storage, SEM: Standard error of mean, SL: Storage length, ST: Storage temperature, TEWL: Total egg weight loss.

*: P < 0.05, ***: P < 0.001, -: P > 0.05.

Descriptive statistics of hatching weight and length of chukar eggs and their effects on different storage length and temperature are given in Table 3. There was no significant difference in chick weight between 7 and 14 days of storage groups. Lower chick weight was observed (P < 0.01) and incubation period was lengthened (P < 0.001) for chukar eggs stored at 15 °C. The incubation period increased when the storage period increased from 7 to 14 day (P < 0.05). No significant interaction was observed among the fixed factors (storage length × temperature) for hatching weight and incubation time.

**Table 3:** Hatching weight and length of chukar eggs under different storage length and temperature.

SL (d)	ST (°C)	n	Chick weight at hatch (g)	Incubation length (d)
**7**	**3**	44	14.50 ± 1.09	23.23 ± 0.42
	**9**	43	14.39 ± 1.27	23.19 ± 0.66
	**15**	40	13.85 ± 1.07	23.75 ± 0.44
	**21**	39	14.66 ± 1.10	23.18 ± 0.39
**14**	**3**	40	14.13 ± 0.93	23.48 ± 0.64
	**9**	44	14.39 ± 1.01	23.43 ± 0.62
	**15**	41	13.81 ± 1.25	23.66 ± 0.48
	**21**	41	14.31 ± 1.18	23.34 ± 0.48
**7**		166	14.35	23.34
**14**		166	14.16	23.48
**SEM**			0.09	0.04
**P-value**			–	*
	**3**	**84**	14.32^a^	23.35^b^
	**9**	**87**	14.39^a^	23.31^b^
	**15**	**81**	13.83^b^	23.70^a^
	**21**	**80**	14.49^a^	23.26^b^
**SEM**			0.12	0.06
**P-value**			**	***
**SL (d) × ST °C)**			–	–

SEM: Standard error of means. SL: Storage length. ST: Storage temperature.

^a.b^Means along the same column with different superscripts are significantly different (*: P < 0.05. **: P < 0.01. ***: P < 0.001. -: P > 0.05).

The effect of storage temperature on hatching performance and embryonic death in chukar eggs stored for either 7 or 14 days is shown in Table 4. No significant differences were reported for the hatching performance. As for embryonic mortality, only embryonic deaths at middle stage were found to be higher for eggs stored at 21 °C for 7 days (P < 0.05).

**Table 4:** The effect of storage temperature on hatching performance and embryonic mortality rate of chukar partridge eggs stored for either 7 or 14 days.

SL (d)	ST (°C)	n	HOES	HOFE (%)	Embryonic Mortality Rate				
					E	M	L	P	Total
**7**	**3**	50	88.00	100.00	0.00	0.00^b^	0.00	0.00	0.00
	**9**	50	86.00	91.49	2.13	0.00^b^	2.13	4.26	8.51
	**15**	48	83.33	88.89	0.00	4.44^ab^	4.44	2.22	11.11
	**21**	49	81.25	84.78	2.17	8.70^a^	2.18	2.17	15.22
**P-value**			–	–	–	*	–	–	–
**14**	**3**	50	80.00	88.89	0.00	2.22	8.89	0.00	11.11
	**9**	49	89.80	89.80	0.00	2.04	8.16	0.00	10.20
	**15**	49	83.67	85.42	0.00	4.17	8.33	2.08	14.58
	**21**	48	85.42	91.11	0.00	0.00	4.44	4.44	8.88
**P-value**			–	–	–	–	–	–	–

E: Early. HOES: Hatchability of the eggs set. HOFE: Hatchability of the fertile eggs L: Late. M: Middle. P: Pipped. SL: Storage length. ST: Storage temperature.

a.b Means along the same column with different superscripts are significantly different *: P < 0.05. -: P > 0.05).

The effect of storage length on hatching performance and embryonic mortality for each storage temperature treatment in chukar eggs is presented in Table 5. Hatchability of the fertile eggs is significantly dropped when storage period lengthened to 14 days in chukar eggs stored at 3 °C (P < 0.05). This may be due to statistically higher embryonic deaths at the late stage (P < 0.05). Apart from these results, no significant differences were observed among the treatment groups.

**Table 5:** The effect of storage length on hatching performance and embryonic mortality rate of chukar partridge eggs stored at different temperatures.

ST (°C)	SL (d)	n	HOES (%)	HOFE (%)	Embryonic Mortality Rate				
					E	M	L	P	Total
**3**	**7**	50	88.00	100.00	0.00	0.00	0.00	0.00	0.00
	**14**	50	80.00	88.89	0.00	2.22	8.89	0.00	11.11
**P-value**			–	*	–	–	*	–	*
**9**	**7**	50	86.00	91.49	2.13	0.00	2.13	4.26	8.51
	**14**	49	89.80	89.80	0.00	2.04	8.16	0.00	10.20
**P-value**			–	–	–	–	–	–	–
**15**	**7**	48	83.33	88.89	0.00	4.44	4.44	2.22	11.11
	**14**	49	83.67	85.42	0.00	4.17	8.33	2.08	14.58
**P**			–	–	–	–	–	–	–
**21**	**7**	48	81.25	84.78	2.17	8.70	2.18	2.17	15.22
	**14**	48	85.42	91.11	0.00	0.00	4.44	4.44	8.88
**P-value**			–	–	–	*	–	–	–

E: Early. HOES: Hatchability of the eggs set. HOFE: Hatchability of the fertile eggs. L: Late. M: Middle. P: Pipped. SL: Storage Length. ST: Storage temperature.

*: P < 0.05. -: P > 0.05.

**Fig 1:**
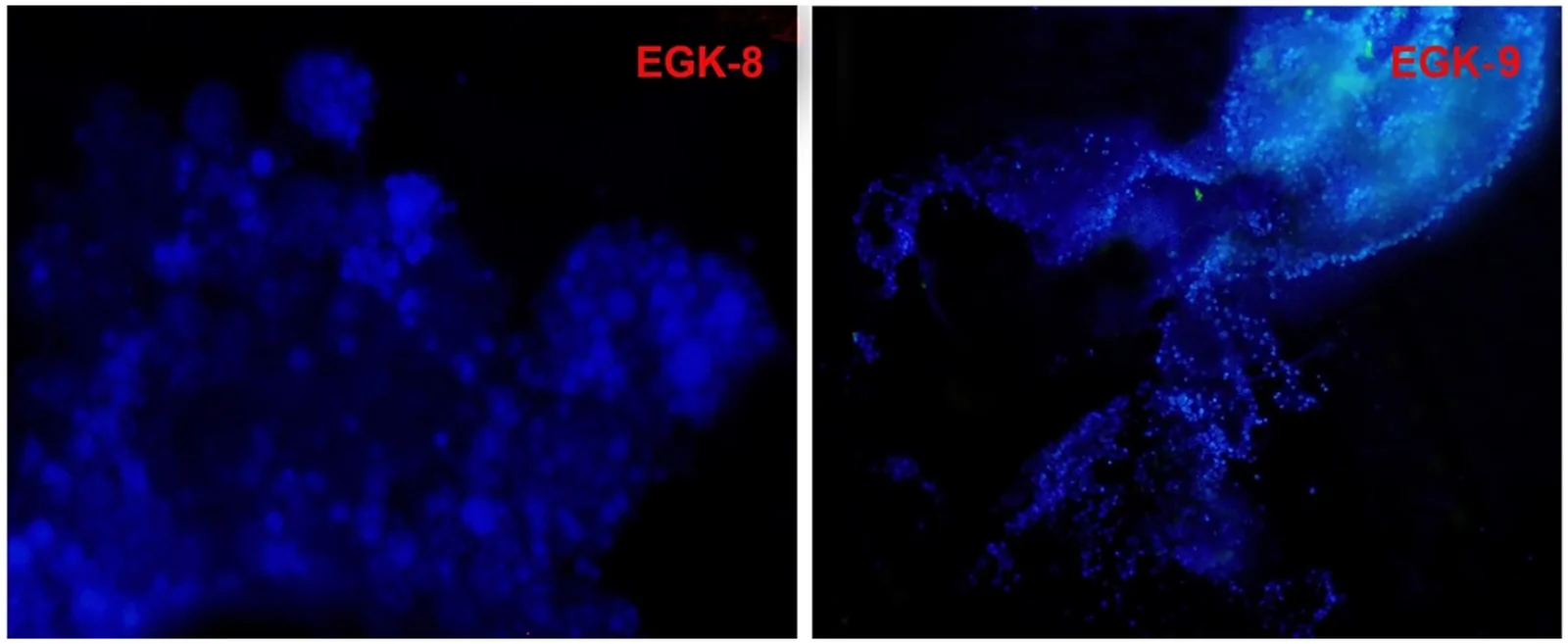
Representative embryonic discs of chukar partridge eggs corresponding to EGK Stage 8 and Stage 9 as defined by Eyal-Giladi and Kochav [14].

Tables 6 and 7 represent the logistic models for the probability of having a higher embryonic development stage and the occurrence of embryonic development (EGK > 10) during storage, respectively. The embryonic development stage did not differ significantly when the storage length was increased from 7 to 14 days. The storage temperature significantly affected the stage of embryonic development in chukar eggs (P < 0.05). The stage of chukar embryos was significantly 2.42 times higher when the temperature was increased from 15 to 21 °C during storage period (P < 0.05). Significant interaction for embryonic stages was observed between storage temperature and length that the chukar eggs stored 7 days at 3 °C showed 68 percent probability of lower stages (P < 0.05). In contrast to the logistic results of embryonic stages, different storage temperatures compared to reference one (15 °C) and storage lengths (14 vs 7 days) didn’t have any significant influence on occurrence of embryonic development (P > 0.05). Morphological evaluation of DAPI-stained embryos confirmed the early pregastrulation stages based on nuclear organization and blastoderm architecture observed under fluorescence microscopy (Fig 1).

**Table 6:** Logistic model for embryonic stages among treatment groups.

Variable	Coefficient	95% CI	SE	OR
**Storage Temperature (°C)**				
**3**	0.08	−0.58, 0.74	0.34	1.08
**9**	−0.28	−0.93, 0.38	0.33	0.76
**21**	0.86*	0.19, 1.58	0.36	2.42
**15**	Reference			
**Storage length (d)**				
**7**	0.57	−0.08, 1.22	0.33	1.77
**14**	Reference			
**Significant Interaction**				
7 d*3 °C	−1.16*	−2.09, −0.23	0.47	0.32

SE: Standard Error. OR: Odds Ratio. CI: Confidence Interval. *: P < 0.05.

**Table 7:** Logistic model for embryonic development occurrence (EGK > 10) during storage among different storage treatments.

Variable^1^	Coefficient	95% CI	SE	OR
**Storage Temperature (°C)**				
**3**	0.13	−0.84, 1.10	0.49	1.14
**9**	0.68	−0.41, 1.77	0.56	1.98
**21**	−0.71	−1.61, 0.20	0.46	0.49
**15**	Reference			
**Storage length (d)**				
**7**	−0.29	−1.20, 0.62	0.47	0.75
**14**	Reference			

SE: Standard Error; OR: Odds Ratio; CI: Confidence Interval.

^1^No significant differences were observed among treatment groups (P > 0.05).

## Discussion

### Weight losses during storage and incubation

During storage, water evaporates from the egg, which may be influenced by both the storage temperature and duration [19]. Water diffusion to the eggshell causes weight loss and therefore an increased air cell during storage [20], which negatively affects the internal quality of eggs and, therefore, hatchability due to moisture loss from albumen during storage [7]. The percentage of partridge egg weight loss during preincubation storage increases with storage time [2,8–10]. Our finding that the rate of egg weight loss during storage increased with storage temperature was in complete agreement with these literatures. Specifically, the weight loss rate during storage was dramatically increased when the storage temperature was increased by 15 °C and above. Similarly, Gomez-de-Travecedo et al [2] reported a greater egg weight loss during storage in red-legged partridges when the storage temperature increased by 15 °C from 9 °C; especially when the storage time was lengthened from 7 d to 42 d. In our study, there was not a significant interaction between storage length and temperature. This may be because the storage length in our experimental design was arranged as 7 and 14 days, the range of which was within the normal storage periods for partridges.

Egg weight loss during incubation is one of the factors affecting hatchability in partridges [21], because too high or too low water loss during incubation was reported to increase embryonic mortality and deteriorate chick quality [22]. The insignificant difference in egg weight loss during incubation on the eggs stored between 7 and 14 days matched well with the literature, which described that the percentage of weight loss during incubation was resistant to time under normal storage duration in partridge eggs [8–10]. Regarding the storage temperature, there was no significant difference or interaction among treatment groups. Gomez-de-Travecedo et al [2] reported a progressive increase in the weight loss of red-legged partridge eggs when the storage temperature was increased from 9 °C to 15 °C.

### Incubation length

Incubation length affects the development rate of chicks and chick uniformity in the poultry industry [23]. The results of the present study agreed with the notion that hatching time is influenced by lengthening the storage period of partridge eggs [2,8]. Storage temperature significantly influenced on the incubation duration when eggs stored at 15 °C were incubated longer compared to other groups. Gomez-de-Travecedo et al [2] reported that storage temperature had no significant effect on the incubation length in red-legged partridges. If the storage time was extended, the eggs would have been stored at lower than 12 °C to obtain optimum hatch window in red-legged partridges.

### Hatchling weight

Chick weight at hatch was mostly affected by the preincubation egg weight and weight loss during incubation [24]. Therefore, storage length had no influence on chick weight at hatch due to the homogeneity of initial egg weights and insignificant differences in weight loss during incubation among the treatment groups. Previous studies of red-legged partridge chicks showed similar results [2]. Çam et al [8] reported statistically different chukar chick weights among storage groups, likely were due to variations in incubation weight loss. Chicks obtained from the eggs stored at 15 °C had the lowest hatched weights, probably due to longer incubation time. In contrast, Gomez-de-Travecedo et al [2] reported that the red-legged chick weight at hatch was not affected by storage temperature.

### Hatching performance and embryonic mortality

Duration and temperature are two crucial factors in storage conditions and are closely related each other [25]. Partridge eggs are recommended to be stored above 12 °C under the condition of normal storage duration [2], which is determined to be until 21 days for optimum hatching results [6–12]. Despite some statistically slight variations in middle or late embryonic mortality rates, the present study revealed that, under the condition of normal storage durations, partridge eggs can be stored from 3 °C to 21 °C without any serious decrease in hatching performance different from the results of Habibi et al [1]. While the ideal temperature for short-term storage is between 12–18 °C in poultry industry, our results revealed that partridge eggs can be stored for short period (< 21 days) at a temperature range of 3–21 °C. This may be because eggs can withstand temperature fluctuations during short-term storage without any significant deterioration in hatching results [26]. However, to our knowledge, this study is the first report that partridge eggs can be stored for normal-term period at 21 °C. This would be a useful tool for the poultry industry, enabling both farmers and hatchery managers to hold partridge eggs for a short period in a room with minimal temperature manipulations.

### Embryonic development

This study showed that the developmental stage of chukar partridge embryos was significantly influenced by storage temperature, while the length of storage did not have a significant impact on either the progression of development or the likelihood of embryos advancing beyond EGK Stage 10. Embryos stored at 21 °C were found to be at more advanced stages compared to those stored at lower temperatures. On average, the EGK stage recorded at 21 °C was approximately 2.4 times higher than that observed at 15 °C. This suggests that 21 °C likely exceeded the physiological zero threshold, allowing embryonic development to continue during storage. These findings are in line with previous those of Özlü et al [15], who demonstrated that broiler embryos were able to develop at 18 °C, especially when temperatures fluctuated between 18 and 21 °C. Similarly, Guinebretiere et al [27] reported that applying thermal stimulation at 18.3 °C during extended storage led to developmental progression up to EGK stages XII to XIII. In contrast, constant storage at 11.6 °C kept the embryos at earlier stages such as EGK X or XI, effectively maintaining them in a state of developmental arrest. Our results are consistent with the staging system proposed by Eyal-Giladi and Kochav [14], which categorizes the earliest phases of avian embryogenesis into 14 morphological stages, leading up to primitive streak formation.

Despite having higher probability of developmental stages in eggs stored at 21 °C, the nonsignificant logistic results of both embryonic development occurrence and hatching performance indicate that most embryos that were stored at or below 21 °C remained in the pregastrulation phase, typically not progressing beyond EGK Stage 10. This pattern suggests that storing the partridge eggs from 3 °C to 21 °C is suitable for maintaining embryos in a stable, arrested state under normal storage length conditions. Embryos stored at 3 °C for seven days were much more likely to remain in early developmental stages, with approximately 68 percent more likely staying at or below EGK Stage 10. In an earlier study, Bakst and Gupta [28] found that low storage temperatures slightly limited embryonic development in turkey eggs but did not increase mortality, which may reflect differences in thermal tolerance between species. Fasenko et al [29] similarly reported that preincubation embryonic development in chicken eggs stored at 14 °C for up to 21 days did not show significant advancement. However, they noted an increase in embryonic mortality with prolonged storage, likely due to increased water loss from the eggs. Although the low temperature effectively stabilized the developmental stage in our study, storing the eggs in refrigerated temperature may not be practical due to possible increased energy costs and required cold storage chambers, considering the insignificant main hatchability results.

## Conclusion

Under normal-term storage period, partridge eggs can be stored from 3 to 21 °C without any serious adverse effects. The results indicate that storing chukar partridge eggs at temperatures between 3 and 21°C for up to two weeks may offer a practical compromise during normal storage duration. The insignificant results for both hatchability and embryonic occurrence in eggs stored at 21 °C suggest that partridge breeders and game farmers might store their eggs during the short-term storage period by placing them in a room with minor temperature and humidity modifications. In addition, storing partridge eggs at higher temperatures would incur less energy costs, thereby decreasing energy demand. Further studies are needed to clarify the high storage temperature effects on partridge eggs.
