# Supplementary material for: Large variability of preincubation storage temperature in chukar partridge eggs under normal storage duration: Impact on egg and hatching traits, and embryonic development
Source: PLoS One. 2026 Sep 23;21(9):e0354754. doi: 10.1371/journal.pone.0354754 (PMC13600485; doi:10.1371/journal.pone.0354754)
Supplement: Supplementary file 2 [file pone.0354754.s001.docx]

**Evaluation of the Protective Effects of *Crocus sativus L.* against Cypermethrin Induced Reproductive Toxicity in Male Rats through the Nrf2 pathway and *In Silico* ADMET analysis**

**Abstract**

Epidemiological studies have demonstrated that prolonged exposure to insecticides such as cypermethrin (CYP) significantly increases the risk of male reproductive toxicity. *Crocus sativus L.* has been recognized for its therapeutic properties, yet its precise role and underlying mechanisms in CYP-induced reproductive dysfunction remain unclear. In this study, total 36 male albino rats were divided into six groups (n=6 per group). CYP (60 mg/kg body weight) was administered in all rat except negative control for 14 days to induce reproductive toxicity. Group 2 served as the CYP-toxicity control, while Group 3 was treated with leuprolide acetate. After toxicity induction, the rats of groups 4 and 5 were treated with *Crocus sativus L.* ethanolic and aqueous extracts at a dose rate of 50 mg/kg body weight, while group 6 received 2.5 ml oral gavage (20 ml/kg b.w.) of *Crocus sativus L.* oil for two weeks (days 15 to 28). The finding indicated that exposure of CYP markedly increased oxidative stress, disrupted testicular architecture, and significantly reduced testosterone levels (P<0.05). Mechanistically, treatment with *Crocus sativus L.* effectively ameliorated these alterations by upregulating Nrf2 expression, restoring antioxidant enzyme activity, and improving testicular histomorphology. Interestingly, molecular docking confirmed strong binding affinities of *Crocus sativus L.* phytoconstituents, including gallic acid, cinnamic acid, and quercetin, to the Nrf2–Keap1 complex. Notably, *Crocus sativus L.* demonstrated significant protective effects against CYP-induced repro-toxicity in male rats, mediated by Nrf2 pathway activation, oxidative damage mitigation, and favorable ADMET properties. Collectively, this study offers a natural, safe, and more effective approach for reproductive-related novel drug development; however, further research is required for validation and clinical application.

**Key Words:**

*Crocus sativus L.*, Cypermethrin, Male Reproductive toxicity, Nrf2 pathway, *In silico* ADMET analysis

**Introduction**

Infertility, a prevalent and emotionally challenging medical condition, has become a subject of increasing concern worldwide, affecting individuals and couples who aspire to conceive (Zhu et al., 2022). World Health Organization (WHO) defines infertility as the inability to become pregnant within a period of one year of normal and unprotected sexual intercourse (Szamatowicz and Szamatowicz, 2020). It is a major health issue that impacts 8–12% of couples of reproductive age globally (Patel et al., 2019). Particularly, male infertility is the cause of 30–50% of the infertility cases, such as abnormal spermatogenesis, hormonal disorders, oxidative stress, and environmental exposures have all played a role in the deterioration of sperm quality and reproductive potential (Jiang et al., 2017; Yan et al., 2015). The growing evidence of a decrease in semen quality has increased public concern in understanding the underlying causes and developing effective treatment for male infertility (Meli et al., 2020; Szamatowicz and Szamatowicz, 2020).

Environmental pollutants, specially pesticides, have been notorious as important contributors to male reproductive problem (Gautam et al., 2024). Pesticides play a critical role in controlling pests and increasing crop production, but they are very dangerous to human beings (Dutta et al., 2023; Oirdi et al., 2024). Among these pesticides, CYP is being widely used as synthetic pyrethroid insecticide, due to its broad-spectrum pest control capabilities (Farag et al., 2021). However, its widely use in agriculture has raised serious concerns about its adverse effects on human reproductive health (Farag et al., 2021). Previous researches have reported that CYP exposure can cause hormonal imbalance, induce oxidative stress, spermatogenesis and reduced fertility (Seven et al., 2022). These adverse effects are primarily linked to the induction of oxidative stress, which disrupts the antioxidant defense mechanisms (Zhang et al., 2021). Importantly, to counter oxidative damage and restore cellular homeostasis, the nuclear factor erythroid 2-related factor 2 (Nrf2) pathway has emerged as a critical target for regulation of cellular antioxidant responses (Aziz et al., 2023; Tantengco et al., 2021). As, CYP has wide negative impact on the nervous, hepatic, renal, and reproductive systems in animals, urging the need for effective therapeutic interventions is important to mitigate its toxicity (Jabbar, 2022).

Interestingly, *Crocus sativus L.* containing natural compounds with antioxidant potential, have shown promise in mitigating chemical induced toxicity. Saffron, as rich in bioactive compounds such as safranal, crocin, and picrocrocin has presented potent antioxidant, anti-inflammatory, and cytoprotective effects (Abdullaev, 2006; Sonay, 2025; Sonay et al., 2024). Due to these properties saffron as a potential candidate for alleviating CYP-induced reproductive damage through modulation of the Nrf2 pathway and reduction of oxidative stress (Arain et al., 2024; Rehman et al., 2025).

Taken together, this study first conducts an in vitro analysis using High-Performance Liquid Chromatography (HPLC) and Gas Chromatography-Flame Ionization Detector (GC-FID) to characterize *Crocus sativus L.* . Mechanistically, the study employs computational approaches such as ADMET (absorption, distribution, metabolism, excretion, and toxicity) to evaluate the pharmacokinetic and safety profiles of its bioactive compounds. Notably, in vivo research investigated the protective effects of *Crocus sativus L.* ethanol extract against CYP-induced reproductive toxicity in male albino rats. This study contributes to the development of safe and effective natural therapies for addressing male infertility and mitigating the adverse effects of environmental toxins on reproductive health.

***Material and Methods***

***2.1 Preparation of*** *Crocus sativus L.* ***Extract***

The *Crocus sativus L.* (saffron) stigmas were procured from the Faisalabad market and were identified from the department of botany at University of Agriculture Faisalabad. For preparation of extract, the saffron stigmas were first air dried in the shade and then finely ground. During the maceration extraction, 10 grams of the dried stigmas were mixed with 100 mL of 96% ethanol in 1:10 ratio as described by Kyriakoudi et al.,(2012) (Kyriakoudi et al., 2012). After that mixture was properly stirred and kept covered for seven days to enhance the extraction process. By using vacuum evaporator the extract was cleaned, purified, concentrated, and stored at 2 to 6 °C in a refrigerator.

***2.2 Chemical and Reagents***

A standard drug Leuprorelin (Leuprolide acetate), was procured form Novartis Pharmaceutical Corporation through Care Pharmacy, Lahore. ELISA kits, acetonitrile and ethanol were purchased form SIGMA ALDRICH (Shanghai, China).

***2.3 Total Phenolic Content*** **of *Crocus sativus L****.* **extracts**

The phenolic content of *Crocus sativus L.* stigma extracts were determined by using Folin Ciocalteu reagent method as described by Baba et al. (2015). The results were expressed in mg of gallic acid equivalent (GAE) per gram of dry sample weight. The experiment was repeated three time for accuracy. During the analysis, a colourimetric assay was used. For that, 0.3 ml of the extract with different concentrations, was mixed with 2.25 ml Folin Ciocalteu’s phenol reagent, 2.5 ml of 6 % sodium carbonate (Na_2_CO_3_) was added an incubated at room temperature for 90 minutes. However, the absorbance of the solution obtained was measured by UV-visible spectrophotometer at wavelength 725 nm. Similarly, the phenolic compounds were measured with reference of a standard calibration curve (0–200 μg/ml) and expressed as mg of gallic acid divide by per gram of dry extract (mg GAE/g DE) (Neupane and Lamichhane, 2020).

***2.4 Total Flavonoid Content of Crocus sativus L.*** ***extracts***

Using the method of Shajari and Moghaddam (2022) the total flavonoid content of *Crocus sativus L.* extracts was measured using colorimetric technique. For obtaining calibration curve stock solution of quercetin with methanol (1mg/ml) was used as standard, however, further diluted to obtain a calibration curve (0-100 µg/ml). For each of the standard and sample solutions, 100 µl was mixed with 25 µl of 1% sodium nitrite and incubated for 5 mins. Additionally, 10 µl of 10% aluminum chloride was further added and incubated again for 5 mins. Finally, 35 µl of 4% sodium hydroxide was added and the mixture was further diluted with 30 µl methanol. By using a spectrophotometer, the absorbance of the resulting solution was measured at a wavelength of 510 nm. The TFC was obtained from the calibration curve equation and converted into milligrams of quercetin equivalent per gram of dry extract (mg QE/g DE) (Shajari and Moghaddam, 2022).

***2.5 Antioxidant Activity and GC-FID Analysis of Crocus sativus L.***

DPPH radical scavenging assay was used for estimation of antioxidant potential of *Crocus sativus L.* extracts. In this method 3ml of *Crocus sativus L.* extracts was combines with 1 ml of 0.004% DPPH solution in methanol. For completion of reaction mixture was kept in dark for 30 min. The absorbance was then measured at 517 nm. The antioxidant capability of ascorbic acid and BHT were evaluated while comparing a drug free solution as control, and test was repeated three times for accuracy. However, the analysis was performed by using Shimadzu equipment such as Pump LC 10AT and UV Visible Detector (Model SPD 10 AV). Similarly, the essential oil of *Crocus sativus L.* was analyzed by using GC FID at Central Hi-Tech Laboratory, University of Agriculture, Faisalabad. The analysis was conducted using a Shimadzu GC-17A system with a DB WAX 30M0.25 column. Nitrogen was used as the mobile phase with a flow rate of 20 ml/min. The temperature program started at 90°C for 1 minute, increased to 180°C at a rate of 2°C per minute, and then to 240°C at a rate of 3°C per minute. The injector temperature was set at 260°C following the method of Anastasaki (Anastasaki et al., 2009).

***2.6 Experimental animals and CYP administration***

In the study, 36 male albino rats weighing 200-250g were purchased from the Animal house of the Department of Microbiology at the University of Agriculture Faisalabad. All rats were housed in well controlled lab settings and had unlimited access to commercial rat pellet food and water. Each of the six groups received six rats at random. The control group had regular food and water, whereas the other five groups (Disease, standard, treatment 1, 2, and 3) received normal food and dose of CYP 60mg/kg body weight for the duration of 14 days. The toxicity was induced by the CYP at dose rate of 60 mg/kg weight in the rats directly by oral gavage for a period of 2 weeks on daily basis (LI et al., 2013). The standard drug Leuprolide acetate (3 mg/kg body weight) 2 Injections was injected intraperitoneally on 15 day and 21 day). After the toxicity induction the rats were treated by saffron ethanolic and water extract at dose rate of 50mg/kg body weight of both extracts for treatment group 1, 2 and 3, while 2.5 ml oral gavage (20 ml/kg b.w.) of saffron oil for 2 weeks days after the induction of toxicity.

***2.7 Sample collection***

At the end of the experiment, the rats were put under anesthesia using 3% sodium pentobarbital (at a dose of 45 mg/kg) and then decapitated. We centrifuged the blood at 4°C for 10 minutes at 800 g. The serum we collected was stored at -80°C for analyzing sex hormones later on. We quickly removed the unilateral testis, the epididymis, and the seminal vesicles, rinsed them with 0.9% saline, and dried them with a paper towel to measure their relative weight. Once weighed, the right testis was frozen at -80°C for counting testicular sperm and measuring daily sperm production. We also took a part of the left testis for histopathological analysis.

***2.8 Weighing of body weight***

The male albino rats were taken out of their cages and put in a plastic container with a lid. The rats were then put on a digital weighing scale and their body weight were list down accordingly for start and end of trial for all the experimental rats.

***2.8 Biochemical analysis***

For the analysis of Testosterone and luteinizing hormone (LH) fresh blood was taken from all experimental rats. Blood collected in gel clot vials and was spun at 3000rpm for about 5 minutes to separate the serum. Analysis is carried out by using the radioimmunoassay technique. Additionally, to assess the antioxidant biomarkers in serum, such as the concentration of SOD, MDA, CAT, GPx in plasma the ELISA was used accordance with the instructions provided in the kits from Nanjing Jiancheng Bioengineering Institute, China.

***2.9 Gene Expression Analysis qRT-PCR***

For gene expression analysis, testis tissues were collected from each group and analyzed using Real Time qPCR. Specific primers were used for amplification of target genes (Table 1). According to the method previously described by Buzdar et al. (2025), cDNA was synthesis was performed followed by RNA extraction (Buzdar et al., 2025). For RNA extraction, 2-3 g of testis tissue samples were homogenized using TRIzol reagent and liquid nitrogen (Win Biosciences, Beijing, China).

For cDNA synthesis, a commercially available TransGen cDNA kit (Biotech Co., Ltd., Beijing, China) was used according to the instructions of manufacturer. A reaction mixture of 20 µL, containing RNA, 2 × TS reaction mix, and oligo (dT) 18 was prepared. The Gene expression of Nrf2, Bax, Bcl-2, Caspase-3, caspase-9, cytochrome-c was determined using a SYBR Green I real time fluorescent quantitative PCR system. Amplification was performed with the TransStart green qPCR SuperMix kit (Biotech, Beijing, China) and each reaction was conducted in triplicate. The amplification protocol consisted of three main steps: denaturation (94°C for 30 s), annealing (61°C for 35 s), and Extension (72°C for 30 s). Relative quantification of each gene was carried out using the comparative 2−ΔΔCT technique.

***2.10 In silico ADMET analysis and Molecular Docking***

For determination of pharmacokinetic properties such as absorption, distribution, metabolism, and excretion (ADMET), the active constituents of *Crocus sativus L.* retrieved using online resources (<http://www.swissadme.ch>) and (<http://biosig.unimelb.edu.au/pkcsm/prediction>). Additionally, for molecular docking, the targeted protein KEAP1-NRF2 (PDB ID: 6QMC) retrieved from an online databank. The protein was prepared using the Protein Preparation Wizard of Maestro 12.5. The optimized protein was then processed for next step. Furthermore, the phytoconstituents of *Crocus sativus L.* were prepared as ligands using the LigPrep tool of Maestro 12.5 software. Docking was performed using Ligand Docking module of Maestro 12.5 to determine proper binding affinities, conformations, and orientations of the ligands with the KEAP1-NRF2 active site (Safdar et al., 2024). The minimized and optimized protein structure of protein was again used; the crystal ligand present in protein was removed and receptor grid was generated. Finally, the ligands were docked properly in to the active pocket of protein. The 2D and 3D interactions were visualized using BIOVIA Discovery Studio Client 2021 and PyMOL software (Buzdar et al., 2025).

***2.11 Histopathological Analysis***

The detailed histopathological procedure for tissue collection and staining began with tissue fixation in 10% buffered formalin for 6-8 days, followed by overnight washing in running water. Tissues were sliced into 4-6μm mm pieces and dehydrated using graded alcohol concentrations (70%, 30%, and 60%). The alcohol was cleared with xylene, and the testicular tissues were embedded in liquid paraffin. Later on, the tissue blocks were prepared using steel and plastic cassettes and cooled at -4°C. The sections were made with help of microtome (RWD Life Science Co., LTD Manufacturer) at 4-5 µm thickness and placed at water bath at 48°C. The sectioned tissue ribbons were mounted on slides, and then stained using H&E staining process. After staining, the slides were fixed with DPX solution and observed under light microscope at 40 X (Chen et al., 2024).

***2.12*** ***Statistical Analysis***

The data ware analyzed using GraphPad Prism 8.0, and the results are presented as mean ± standard deviation (mean ± SD). Group comparisons were performed using one-way ANOVA followed by Tukey’s post hoc test. A *p*-value of less than 0.05 was considered as statistically significant.

***Results***

***3.1 Evaluation of Phytochemicals present in Crocus sativus L.*** ***extract with HPLC***

The extracts of *Crocus sativus L.* were analyzed using high-performance liquid chromatography (HPLC), and the chromatogram (Figure 1) shows the different compounds presents in the extract. In Table 1, among the detected compounds, quercetin showed highest concentration (304.86 ppm, RT 2.89), followed by Kaempferol (254.98 ppm, 2.49), and Vitamin C (59.34 ppm, RT 3.96). Other notable constituents included Limonin (18.387 ppm, RT 91.3), Gallic acid (9.15 ppm, RT 4.513), gamma-undecalacetone (4.087 ppm, RT 8.7), Vanillic acid (1.14 ppm, RT 13.6), benzoic acid (0.85 ppm, RT 14.98), Cinnamic acid (0.25 ppm, RT 25.27), and Chlorogenic acid (0.06 ppm, RT 15.66). Figure 1 (a-d) further supported the chromatographic profile, indicated that phenolic compounds, flavonoids and vitamin C were the dominant constituents of the extract highlighting the rich antioxidant potential of *Crocus sativus L. .*

**Table 1: Identified retention time and concentration of components in *Crocus sativus L.*** **extract under gradient of HPLC analysis**

| **Compounds** | **Structure** | **Retention time** | **Concentration (ppm)** |
| --- | --- | --- | --- |
| Quercetin |  | 2.893 | 304.86 |
| Gallic acid |  | 4.513 | 9.15 |
| Vanillic Acid |  | 13.600 | 1.14 |
| Benzoic acid |  | 14.987 | 0.85 |
| Chlorogenic Acid |  | 15.660 | 0.06 |
| Cinnamic Acid |  | 25.273 | 0.25 |
| Kaempferol |  | 2.490 | 254.98 |
| Vitamin C |  | 3.960 | 59.34 |
| gamma-undecalacetone |  | 8.7 | 4.087 |
| Limonin |  | 91.3 | 18.387 |

**Figure 1:** Graphs represent chemical compounds of *Crocus sativus L.* HPLC results for the a) phenolic compounds b) Kaempferol c) Vitamin C and d) GC fid analysis of compounds presents in *Crocus sativus L.*

***3.2 Phytochemicals in*** *Crocus sativus L.* ***extract detected by GC fid analysis compounds***

Gas chromatography with flame ionization detection (GC–FID) analysis of *Crocus sativus L.* extract identified several phytochemical constituents with varying concentrations and retention times (Table 2). The most abundant compounds were tripropylene glycol (29.74%, RT 11.16) and trimethyl propane (29.69%, RT 11.35), followed by triethylene glycol monobutyl ether (7.71%, RT 11.68), 3-hydroxynonanoic acid (7.56%, RT 11.57), and methyl linoleate (10.04%, RT 11.95). Other detected compounds included trimethylolpropane (5.31%, RT 11.77), methyl oleate (4.52%, RT 12.30), triethylene glycol (2.30%, RT 11.47), and methyl stearate (1.34%, RT 38.95). Minor constituents comprised heneicosylic acid (0.54%, RT 38.77; 0.07%, RT 44.68), hentriacontylic acid (0.80%, RT 44.00), paullinic acid (0.18%, RT 39.13), motanic acid (0.12%, RT 44.18), and methyl erucate (0.08%, RT 39.76). The GC–FID profile highlighted the predominance of glycol derivatives and fatty acid esters, suggesting a diverse phytochemical composition that may contribute to the biological activities of *Crocus sativus L..* Figure 2 supported the GC-fid profile, of the dominant constituents of essential oil of *Crocus sativus L..*

**Table 2. Identified retention time and concentration of components in *Crocus sativus L.* extract under GC fid analysis**

| **Compounds** | **Structure** | **Concentration%** | **Retention time** |
| --- | --- | --- | --- |
| Tripropylene glycol |  | 29.74 | 11.163 |
| Trimethyl propane |  | 29.69 | 11.350 |
| Triethylene glycol |  | 2.30 | 11.467 |
| 3- Hydroxynonanoic acid |  | 7.56 | 11.579 |
| Triethylene glycol monobutyl ether |  | 7.71 | 11.684 |
| Trimethylolpropane |  | 5.31 | 11.769 |
| Methyl linoleate |  | 10.04 | 11.951 |
| Methyl Oleate |  | 4.52 | 12.298 |
| Heneicosylic acid |  | 0.54 | 38.770 |
| Methyl Stearate |  | 1.34 | 38.950 |
| Paullinic acid |  | 0.18 | 39.133 |
| Methyl erucate |  | 0.08 | 39.761 |
| Hentriacontylic acid |  | 0.80 | 44.003 |
| Motanic acid |  | 0.12 | 44.187 |
| Heneicosylic acid |  | 0.07 | 44.680 |

**Figure 2:** Graphical curve showing the concentration of phytoconstituents from essential oil of *Crocus sativus L.* analyzed by GC-fid

***3.3 Total phenolic, total flavonoid and antioxidant potential of ethanol and aqueous extract of Crocus sativus L.***

Table 3 represents the quantified total phenolic content (TPC), total flavonoid content (TFC), and antioxidant potential of *Crocus sativus L.* extracts. The results revealed that *Crocus sativus L.* ethanolic extract contain significantly (P<0.05) higher concentration of phytoconstituents and antioxidant capability compared to the aqueous extract. As the ethanolic extract of *Crocus sativus L.* shown a TPC of 8.19 ± 0.05 mg GAE/g and TFC of 8.19 ± 0.05 mg QE/g. But the aqueous extract of *Crocus sativus L.* exhibit a lower concentration 6.49 ± 0.04 mg GAE/g and 3.53 ± 0.08 mg QE/g, respectively. Similarly, antioxidant potential measure by DPPH radical scavenging activity was significantly (P<0.05) higher in ethanolic extract 101.60 ± 2.5%) compared to the aqueous extract (91.28 ± 1.95%).

**Table 3: Total Phenolic Contents, Total Flavonoid Contents, and antioxidant potential of ethanol and aqueous extract of *Crocus sativus L.***

| **Nature** | **Ethanol extract** | **Aqueous extract** |
| --- | --- | --- |
| **TPC** | 8.19 ± 0.05 | 6.49±0.037 |
| **TFC** | 8.19 ± 0.05 | 3.53±0.08 |
| **%DPPH** | 101.60±2.5 | 91.28±1.95 |

***3.4 Pharmacokinetic and Toxicity Profiles of Phytochemicals of Crocus sativus L.***

The pharmacokinetic and toxicity profiling of phytochemicals extracted form *Crocus sativus L.*, including quercetin, gallic acid, vanillic acid, benzoic acid, chlorogenic acid, cinnamic acid, and kaempferol, revealed promising pharmacokinetic properties such as absorption, distribution, metabolism, and excretion that may underpin their therapeutic potential. Among these, benzoic acid and cinnamic acid exhibited in excellent intestinal absorption (100% and 94.83% respectively) and high Caco2 permeability, suggest efficient systemic uptake, whereas chlorogenic acid showed limited absorption capacity.

Distribution analysis indicated that quercetin and kaempferol possessed higher good steady-state volumes (1.56 and 1.27 log L/kg), implying broader tissue availability that increase biological activity, while gallic and vanillic acids showed restricted tissue distribution. Cinnamic acid displayed the highest potential for blood brain barrier penetration (0.45 log BB), suggests possible neuroprotective roles.

Moreover, the rate of clearance ranged chlorogenic acid (0.31) and cinnamic acid (0.78), supporting moderate systemic retention conductive to sustained pharmacological effects. Similarly, toxicological predictions indicated overall safe with no sign of mutagenicity, hepatotoxicity and skin sensitization, and low chronic toxicity value. Therefore, the findings presented in Table 4, suggest that phytochemicals of *Crocus sativus L.* demonstrate favorable pharmacokinetic properties with minimal toxicity, though variability in absorption and tissue distribution may influence their therapeutic potential.

**Table 4:** ***In silico* ADMET analysis of phytochemical of *Crocus sativus L.*** **using swissadme and pkCSM tool**

| **Pharmacokinetic Properties** | | **Selected Phytochemicals** | | | | | | | | |
| --- | --- | --- | --- | --- | --- | --- | --- | --- | --- | --- |
| **Properties** | **Model Name** | **MCF1** | **MCF2** | **MCF3** | **MCF4** | **MCF5** | | **MCF6** | | **MCF7** |
| **Absorption** | **Water solubility** (log mol/L) | -2.93 | -2.56 | -1.84 | -1.74 | -2.45 | -2.61 | | -3.04 | |
|  | **Caco2 permeability (**log Papp in 10^6^ cm/s) | -0.23 | -0.08 | 0.33 | 1.71 | -0.84 | 1.72 | | 0.03 | |
|  | **Intestinal absorption** % | 77.21 | 43.37 | 78.15 | 100 | 36.37 | 94.83 | | 74.29 | |
|  | **Skin Permeability** (log Kp) | -2.74 | -2.74 | -2.73 | -2.73 | -2.74 | -2.69 | | -2.74 | |
|  | **P-glycoprotein substrate** | + | - | - | - | + | - | | + | |
|  | **P-glycoprotein I inhibitor** | - | - | - | - | - | - | | - | |
|  | **P-glycoprotein II inhibitor** | - | - | - | - | - | - | | - | |
| **Distribution** | **VDss (human)** (log L/kg) | 1.56 | -1.85 | -1.74 | -1.64 | 0.58 | -1.05 | | 1.27 | |
|  | **Fraction unbound** (Fu) | 0.21 | 0.62 | 0.52 | 0.52 | 0.66 | 0.38 | | 0.18 | |
|  | **BBB permeability** (log BB) | -1.09 | -1.11 | -0.38 | -0.22 | -1.41 | 0.45 | | -0.94 | |
|  | **CNS permeability** (log PS) | -3.06 | -3.74 | -2.63 | -2.0 | -3.86 | -1.84 | | -2.23 | |
| **Metabolism** | **CYP2D6 substrate** | - | - | - | - | - | - | | - | |
|  | **CYP3A4 substrate** | - | - | - | - | - | - | | - | |
|  | **CYP1A2 inhibitor** | + | - | - | - | - | - | | + | |
|  | **CYP2C19 inhibitor** | - | - | - | - | - | - | | - | |
|  | **CYP2C9 inhibitor** | - | - | - | - | - | - | | - | |
|  | **CYP2D6 inhibitor** | - | - | - | - | - | - | | - | |
|  | **CYP3A4 inhibitor** | - | - | - | - | - | - | | - | |
| **Excretion** | **Total Clearance** (log ml/min/kg) | 0.41 | 0.52 | 0.63 | 0.71 | 0.31 | 0.78 | | 0.48 | |
|  | **Renal OCT2 substrate** | - | - | - | - | - | - | | - | |
| **Toxicity** | **AMES toxicity** | - | - | - | - | - | - | | - | |
|  | **Max. tolerated dose (human)** (log mg/kg/day) | 0.49 | 0.7 | 0.72 | 0.61 | 0.31 | 1.11 | | 0.53 | |
|  | **hERG I inhibitor** | - | - | - | - | - | - | | - | |
|  | **hERG II inhibitor** | - | - | - | - | - | - | | - | |
|  | **Oral Rat Acute Toxicity (LD50)** (mol/kg) | 2.47 | 2.22 | 2.45 | 2.17 | 1.97 | 2.09 | | 2.45 | |
|  | **Oral Rat Chronic Toxicity (LOAEL)** (log mg/kg_bw/day) | 2.61 | 3.06 | 2.03 | 2.64 | 2.98 | 2.65 | | 2.51 | |
|  | **Hepatotoxicity** | - | - | - | - | - | - | | - | |
|  | **Skin Sensitization** | - | - | - | - | - | - | | - | |
|  | ***T. Pyriformis* toxicity** (log ug/L) | 0.28 | 0.28 | 0.26 | 0.08 | 0.28 | 0.25 | | 0.31 | |
|  | **Minnow toxicity** (log mM) | 3.72 | 3.18 | 1.93 | 1.84 | 5.74 | 1.72 | | 2.88 | |

**Note:** MCF1; Quercetin, MCF2; Gallic acid, MCF3; Vanillic Acid, MCF4; Benzoic acid, MCF5; Chlorogenic Acid, MCF6; Cinnamic Acid, MCF7; Kaempferol. Yes = + and No = -

***3.5 Effects of Crocus sativus L. on Body Weight of Rats***

Administration of *Crocus sativus L.* extracts demonstrated a notable influence on the body weight of experimental rats (Table 5). The Mean (± SD) final body weight in the normal group was 219.25 ± 8.62 g, whereas the positive control group showed a significant (P<0.05) decline 205.5 ± 3.32 g. However, rats treated with standard drug Leuprolide acetate recorded 207.75 ± 8.46 g a significant (P<0.05) improvement compare to positive control. Interestingly, the treatment groups received *Crocus sativus L.* exhibited divergent outcomes depending on the extract form. The aqueous extract *(AQE)* group rat showed body weight (201.5 ± 4.51 g) slightly lower than standard group, while the ethanolic extract (EthE) group displayed an improve body weight (209.3 ± 4.27 g). Notably, the oil extracted group achieved the highest final body weight was significantly higher (P<0.05) compared to both normal control and positive control groups. The findings indicate that oil fraction of *Crocus sativus L.* exerts the most pronounced positive effect on body weight restoration and mitigate the toxic effect of CYP.

***3.6 Effects of Crocus sativus L.*** ***on Gonadal of Male Rats***

The weights of the testes and epididymis (Table 5) were also assessed to evaluate the potential reproductive benefits of *Crocus sativus L.*. In the normal control group, the weight of (testes) was 3.63 ± 0.11 g, whereas a marked reduction was observed in the positive control group (2.78 ± 0.16 g, p<0.05). The standard drug Leuprolide acetate group significantly (P<0.05) restored the gonadal weight to 3.44 ± 0.21 g. However, treatment with *Crocus sativus L.* extracts remarkably improved the toxic effect of CYP. The AQE group recorded 3.45 ± 0.17 g, while the EthE group showed the highest gonadal weight (3.66 ± 0.17 g), exceeding, even the normal control values. Similarly, the oil-treated group demonstrated a significant recovery, with a gonadal weight of 3.48 ± 0.15 g. These findings indicate that both the ethanolic and oil extracts of *Crocus sativus L.* are effective in restoring testicular and epididymal weight, suggesting a potential role in the preservation and improvement of male reproductive health against CYP-induced toxicity.

**Table 5:** Rats initial weight, final weight, and weight of testes and epididymis.

|  | **Normal Control** | **Positive Control** | **Standard** | ***Crocus sativus L. (AQE)*** | ***Crocus sativus L.***  **(EthE)** | ***Crocus sativus L.***  **(oil)** |
| --- | --- | --- | --- | --- | --- | --- |
| **Initial weight of rats** | 114.750 ± 9.878 | 121.250± 13.59* | 119.500± 11.121*# | 113..250± 6.397#$ | 118.500± 16.422*# | 117.750± 11.58 #$ |
| **Final weight of rats** | 219.250± 8.617 | 205.500± 3.317* | 207.750± 8.461# | 201.500± 4.509*$ | 209.250± 4.272* | 231.000± 10.893 *#$ |
| **Testes and epididymis weight** | 3.628 ± 0.105 | 2.778 ± 0.163* | 3.443 ± 0.203*# | 3.448± 0.170*#$ | 3.660± 0.171#$ | 3.478 ± 0.14 *# |

**Note:** **p* < 0.05 indicates a significant difference compared with the control group;
\#*p* < 0.05 indicates a significant difference compared with the CYP-treated group;
$*p* < 0.05 indicates a significant difference compared with the standard control group.
Values are expressed as mean ± SD (n = 6).

***3.7 Effects of Crocus sativus L.*** ***on the serum level of sex hormones of rats***

Administration of *Crocus sativus L.* exert beneficial effect the serum levels of sex hormones in rats (Table 6). Compared to the positive control group, which showed a marked reduction in testosterone (4.86 ± 0.64 ng/mL) and luteinizing hormone (2.44 ± 0.49 mIU/mL), treatment with aqueous extract, ethanol extract, and oil of *Crocus sativus L.* resulted in notable improvements. Testosterone levels increased to 11.84 ± 1.34, 12.63 ± 1.08, and 12.67 ± 1.36 ng/mL, respectively, while luteinizing hormone levels rose to 5.23 ± 0.72, 5.30 ± 0.42, and 6.19 ± 1.55 mIU/mL. Similarly, spermatocyte counts, which had declined in the positive control group (34.23 ± 0.08 million/mL), were restored toward normal with values of 46.38 ± 0.13, 48.58 ± 0.09, and 44.62 ± 0.07 million/mL, respectively.

**Table 6: Effect of *Crocus sativus L.* exposure on serum level of sex hormones on rats**

|  | Normal Control | Positive Control | Standard | *Crocus sativus L. (AQE)* | *Crocus sativus L.* (EthE) | *Crocus sativus L.* (oil) |
| --- | --- | --- | --- | --- | --- | --- |
| **Testosterone** | 19.32 ± 0.528 | 4.86 ± 0.64* | 17.77± 11.53 # | 11.84± 1.34 *#$ | 12.63 ± 1.08 *#$ | 12.67 ± 1.36 *#$ |
| **Luteinizing** | 6.055 ± 0.884 | 2.438 ± 0.494* | 6.53 ± 0.457 | 5.225 ± 0.724# | 5.303 ± 0.417 *#$ | 6.185 ± 1.548 # |
| **Spermatocytes** | 46.530 ± 0.183 | 34.230 ± 0.075* | 45.468 ± 0.299# | 46.375 ± 0.129# | 48.578 ± 0.090 *#$ | 44.618 ± 0.072# |

**Note:** **p* < 0.05 indicates a significant difference compared with the control group;
\#*p* < 0.05 indicates a significant difference compared with the CYP-treated group;
$*p* < 0.05 indicates a significant difference compared with the standard control group.
Values are expressed as mean ± SD (n = 6).

***3.8 Effects of Crocus sativus L.*** ***on activity of Antioxidant Enzyme concentration***

Figure 3 (a-d), illustrates the serum concentrations of oxidative stress markers such as superoxide dismutase (SOD), Malondialdehyde (MDA), catalase (CAT), and Glutathione peroxidase (GSH-px), assessed in this study. A significant (p<0.05) reduced in the activity of antioxidant enzymes was observed in CYP treated group. However, treatment with the ethanolic, aqueous extract and oil of *Crocus sativus L.*, markedly improved the activity levels of these enzymes. Conversely, the CYP treated group showed a significant (p<0.05) elevation in MDA concentration, while treatment with *Crocus sativus L.* led to a substantial reduction. Moreover, SOD, CAT and GSH-px levels were restored to near normal values in the *Crocus sativus L.* treated groups, indicating an effective normalization of oxidative stress in the rats. Importantly, these findings revealed that *Crocus sativus L.,* in both extract and oil forms, serves as an efficient agent in reducing oxidative stress and preserving cellular integrity.

**Figure 3:** Changes in antioxidant enzyme levels in the testis and blood of male rats (n = 6) orally administered different doses of *Crocus sativus L.* for 28 days. Statistical analysis was performed using one-way ANOVA followed by Tukey’s post hoc test. Values are expressed as mean ± SD (n = 6). **p* < 0.05 indicates a significant difference compared with the normal control group; #*p* < 0.05 compared with the CYP-treated group; and $*p* < 0.05 compared with the standard control group. (a) SOD, (b) MDA, (c) CAT, (d) GPx

## ***3.9 Effect of Crocus sativus L. on apoptotic gene expressions of testes tissues of rats***

In this study, CYP induced apoptosis in the testicular tissues of rats, and the gene expression of Nrf2, Bcl2, Cytochrome-c, Caspase-3, Caspase-9, Bax were evaluated in the testes. The gene expression of Nrf2, Bcl2, were significantly up regulated by *Crocus sativus L. (AQE)*, *Crocus sativus L. (*EthE*)* and *Crocus sativus L. (OIL)* compared with CYP treated group in testes in rats. In contrast the level of Bax, Caspase-3, Caspase-9, and Cytochrome-c were downregulated in treated groups *Crocus sativus L. (AQE)*, *Crocus sativus L. (*EthE*)* and *Crocus sativus L. (OIL)* but significantly down in CYP treated groups in testicular tissues of rats (Figure 4 a-f).

**Figure 4: Effect of *Crocus sativus* L. on the mRNA expression of apoptosis-related genes in the testes of male rats.** Data are presented as mean ± SD (n = 6). Statistical analysis was performed using one-way ANOVA followed by Tukey’s post hoc test. *p* < 0.05 indicates a significant difference compared with the normal control group; #*p* < 0.05 compared with the CYP-treated group; and $*p* < 0.05 compared with the standard control group. (a) Nrf2, (b) Bcl-2, (c) Bax, (d) Cytochrome c, (e) Caspase-3, (f) Caspase-9**.**

***3.10 Molecular Docking***

The Keap1-Nrf2 pathway serves as a vital cellular defense mechanism against oxidative and electrophilic stress, with Nrf2 (nuclear factor erythroid 2-related factor 2) acting as a transcription factor that activates detoxifying and protective genes, while Keap1 (Kelch-like ECH-associated protein 1) represses Nrf2 under normal conditions by promoting its degradation. Docking studies represented that natural compounds like Gallic acid, Cinnamic acid, Quercetin, Vanillic acid, Benzoic acid, Kaempferol, and Chlorogenic acid significantly inhibited the Nrf2-Keap1 interaction, demonstrated strong docking scores. The highest docking score was achieved for Gallic acid having binding affinity (-6.95 kcal/mol), followed by Cinnamic acid (-6.93 kcal/mol), Quercetin (-6.62 kcal/mol), Vanillic acid (-5.98 kcal/mol), Benzoic acid (-5.51 kcal/mol), Kaempferol (-5.47 kcal/mol), and Chlorogenic acid (-4.51 kcal/mol) (Figure 5H). Figure 4G representing the redocking of the co-crystallized ligand yielded an RMSD value of 0.2117 Å, indicating excellent reproducibility of the experimental binding pose. This low RMSD value validates the accuracy and reliability of the docking protocol, as the predicted binding conformation closely matches the crystallographic structure presented in the study. Furthermore, the binding affinities below -6 kcal/mol generally indicate a biologically relevant and stable interaction between ligand and receptor. Therefore, the docking results suggest the Gallic acid, Cinnamic acid, and Quercetin may effectively disrupt the Keap1–Nrf2 complex and enhance Nrf2 activation under oxidative stress conditions. The key amino acid residues involved in the active binding site included ARG A: 415, SER A: 508, TYR A: 525, ARG A: 483, PHE A: 478, SER A: 555, and ALA A: 556, with most compounds interacting with similar residues. Notably, Gallic acid showed the most efficient docking by strongly inhibiting the Nrf2-Keap1 protein-protein interaction and forming multiple hydrogen bonds with critical amino acid residues (Figure 5 A–F).

**Figure 5: Molecular docking of phytoconstituents with Nrf2-Keap1 with inhibitory ligand complex and molecular interaction is presented in 3D and 2D diagrams.** A) Represents Gallic acid interaction with Nrf2-Keap1, B) Represents Cinnamic interaction with Nrf2-Keap1, C) Represents Quercetin interaction with Nrf2-Keap1, D) Represents Vanillic acid interaction with Nrf2-Keap1, E) Represents Benzoic acid interaction with Nrf2-Keap1, F) Represents Kaempferol interaction with Nrf2-Keap1, G) Superimposed structure showing the alignment of the redocked co-crystallized ligand with the original crystallographic pose, and H) Represent docking score of compounds

***3.11 Histopathological Analysis***

The histological observations shown no disruption in the spermatogenesis of control group and *Crocus sativus L.* extract treated groups. Have normal seminiferous tubule diameter and germinal epithelium. The histological observations show high disruption in CYP treated group and increase interstitial spaces and diameter of seminiferous tubules. However, Restoration by *Crocus sativus L.* treated group showing restoration of normal spermatogenesis in the majority of seminiferous tubules and normal proliferating germ cells (Figure 6 a-f).

**Figure 6: Histopathological observation of testes of rats treated with *Crocus sativus.*** *a);* Normal Control, b); Positive Control, c); Standard drug, d); *Crocus sativus L. (AQE), e); Crocus sativus L.* (EthE), and (f); *Crocus sativus L.* (oil).

**Discussion**

This study provides compelling evidence that *Crocus sativus L.* and its phytoconstituents confer significant protection against CYP-induced reproductive toxicity in male rats, primarily through modulation of the Nrf2 pathway and enhancement of antioxidant defenses. Comprehensive *in vivo* assessments revealed that both the ethanolic extract and oil fraction of *Crocus sativus L.*, restored body weight, testicular indices, and reproductive hormones (testosterone and luteinizing hormone), thereby mitigating CYP-induced reproductive impairment. Parallel analyses robust antioxidant activity, with treatment groups representing normalization of superoxide dismutase, catalase, and glutathione peroxidase levels, along with a significant reduction in malondialdehyde. At the molecular level, *Crocus sativus L.* upregulated Nrf2 and Bcl-2 expression while downregulating pro-apoptotic markers including Bax, Cytochrome-c, Caspase-3, and Caspase-9, highlighting its potent anti-apoptotic efficacy. *In silico* ADMET analysis further validated the pharmacokinetic properties and safe use of phytochemicals present in *Crocus sativus L. extract*. However, molecular docking showed that gallic acid, cinnamic acid, and quercetin act as strong inhibitors of the Keap1–Nrf2 interaction, promoting sustained antioxidant signaling. Additionally, Histopathological evaluations corroborated these findings, showing restoration of normal spermatogenesis and preserved testicular architecture. Collectively, these results underscore the therapeutic potential of *Crocus sativus L.* as a natural intervention against pesticide-induced male reproductive toxicity.

In term of pharmacokinetic properties, the phytochemicals presented in the *Crocus sativus L.* exhibited significant absorption, distribution, metabolism and excretion (Sonay et al., 2025), which support their therapeutic potential (Butnariu et al., 2022). Consistent with earlier reports by Cong et al. (2001), reported intestinal absorption of benzoic acid at is 92-96 % (Behan et al., 2024; Cong et al., 2001), our findings confirmed complete absorption (100 %), reinforcing its high bioavailability. In contrast, chlorogenic acid showed markedly low absorption 36.37 % compared with the 67 % reported by Lafay et al. (2006) (Lafay et al., 2006), a discrepancy that may be attributed to inter drug interactions, matrix effect or differences in experimental models. Distribution analysis revealed that quercetin exhibited relatively high steady state volume (1.56 log L/kg), closely aligning with the 1–2 log L/kg range reported by Zhang et al. (2023) (Zhang et al., 2023). Similarly, Kaempferol 1.27 log L/kg showed an exact match with findings of Prasetyawan et al. (2025) (Prasetyawan et al., 2025) highlighting consistency across independent studies. Regarding excretion, the clearance rate of (0.31 mL/min/kg) and cinnamic acid (0.78 mL/min/kg) were in agreement with previously reported values (Mykhailenko et al., 2022), further validating the reproducibility of these pharmacokinetic predictions (Bellachioma et al., 2023; Butnariu et al., 2022).

Notably, CYP exposure induced significant reproductive toxicity in male rats, characterized corroborating earlier findings (Li et al., 2023; LI et al., 2013; Abd El-Hameed and Mahmoud, 2020). Importantly the reduction in reproductive organ weight in CYP treated group may attributed to suppressed spermatogenic activity and decreased synthesis of essential reproductive hormones (Poli and Motireddy, 2025). Interestingly, *Crocus sativus L.* co-administration mitigated this toxic effect and reflect therapeutic and restorative effects. In this research the treatment groups, particularly those received the ethanolic and oil fraction of *Crocus sativus L.,* demonstrated substantial improvement in reproductive organ weight and balanced hormones in rats. These results are in accordance with Yari et al. (2016), reported that *Crocus sativus L.* ameliorates cadmium induced reproductive toxicity, and improve body weight and testicular function in male rats (Yari et al., 2016)..

The findings further revealed that CYP caused significant structural damage to the testes, such as reduced seminiferous tubule diameter, decreased seminiferous epithelium height, and increased interstitial spaces, accompanied by marked reduction in plasma luteinizing hormone and testosterone levels (LI et al., 2013; Katragadda et al., 2021). Remarkably, treatment with *Crocus sativus L.* properly reversed these effects and preserve the testicular architecture, restore spermatogenesis, regulate testosterone and LH concentration (Tarradas et al., 2020). Consequently, these protective actions align with previous observation of Moshfegh et al. (2022) reported that *Crocus sativus L.* enhance androgenic activity and improve male reproductive function under toxic stress (Hasheminasab et al., 2024; Li et al., 2025).

Abdus Sallam et al. (2020) reported that CYP exposure in male rats significantly alters various serum biochemical markers, indicating potential toxicity (Abdus Sallam et al., 2020). Importantly, seminiferous tubules and germ cell lines were damaged due to oxidative stress produced by CYP (Zhang et al., 2021). However, it has been observed that *Crocus sativus* extract treatment normalized the serum biochemical markers (Mafi et al., 2025). Such as Memarzia et al. (2023) reported *Crocus sativus* extracts increase the antioxidant enzymes, such as superoxide dismutase (SOD) and catalase (CAT), which reduce oxidative stress by neutralizing free radicals (Memarzia et al., 2023). Additionally, *Crocus sativus* extract protect animals from oxidative stress by boosting antioxidant defense mechanisms and increasing antioxidant state (Zeka et al., 2020). Importantly, CYP induced oxidative stress led to degeneration of germ cells and seminiferous tubules (Zhang et al., 2021).

Connectively, at the gene level, *Crocus sativus L.* mitigate CYP-induced mitochondrial apoptosis by downregulation of Bax, Cytochrome-c, Caspase-3, and Caspase-9 and upregulation of Nrf2 and Bcl-2. This coordinated regulation preserved mitochondrial integrity, reduced apoptotic cell death and supported spermatogenesis, as reported in previous study (Ashraf et al., 2024; Kabeer et al., 2025). The findings of molecular docking further supported, as key phytoconstituents of *Crocus sativus L.* including gallic acid (Chiavaroli et al., 2024), cinnamic acid, and quercetin strongly inhibited the Nrf2–Keap1 interaction, therefore promoting sustained Nrf2 activation and reinforcing the observed *in vivo* therapeutic outcomes (Buzdar et al., 2025). These findings are further strengthened by histopathological analysis, which revealed restoration of normal testicular morphology, improved seminiferous tubule diameter, germinal epithelium thickness, and reduce interstitial spaces. Finally, *Crocus sativus L.* not only modulates gene expression but also preserves structural integrity and spermatogenesis in CYP exposed rats. Previous studies also showed that CYP exposure in testes showed deformed Sertoli cells degenerative damage in seminiferous tubules with loss of spermatogenic cell layers (Bushra and Bastwrous, 2022) but treatment of *Crocus sativus L.* preserve the histological structures (Butnariu et al., 2022; Kandil et al., 2024).

**Conclusion**

The current study concluded that *Crocus sativus L.* extract had a therapeutic effect against CYP reproductive toxicity in male rats through a multifaceted mechanism involving restoration of hormonal balance, enhancement of antioxidant defenses, and regulation of apoptotic gene expression. The phytoconstituents of *Crocus sativus L.*, supported by favorable pharmacokinetic and toxicity profiles, activated the Nrf2 pathway, suppressed pro-apoptotic signaling, and preserved testicular structure and spermatogenesis, as further validated by molecular docking and histopathological analysis. Interestingly, these findings highlight the therapeutic potential of *Crocus sativus L.* as a safe and effective natural intervention for mitigating pesticide-induced reproductive damage, paving the way for its translational application in reproductive health management.
