# Supplementary material for: Large variability of preincubation storage temperature in chukar partridge eggs under normal storage duration: Impact on egg and hatching traits, and embryonic development
Source: PLoS One. 2026 Sep 23;21(9):e0354754. doi: 10.1371/journal.pone.0354754 (PMC13600485; doi:10.1371/journal.pone.0354754)
Supplement: Supplementary file 3 [file pone.0354754.s002.docx]

To reviewer 1:

I’m grateful for your advice and thanks for your efforts to improve the manuscript. I have made a revision according to all of your suggestions below;

Abstact

Abstract:

*1-In line number 32 please add “were” after “the eggs”.*

Done

*2-In line number 35 please change “on” to “in”.*

Done

*3-In line number 38 please change “at from” to “between”.*

Revised.

*4-In line number 41 please change “farmers would storage” to farmers would store”.*

Done

*Introduction:*

*1-In line number 49 please change “valuable” to “valuability”.*

Done

*2-In line number 60 please change “42 d” to “42 days”.*

Done

*3-Please give reference at the end of line number 67.*

No need to give the reference since the sentence belongs to the previous one which was already cited.

*4-In line number 70 please change “under” to “for”.*

Done

*Materials and Methods:*

*1-In line number 82 please add “the” before department.*

Done

*2-In line number 86 please add “was” before “created”.*

Done

*3-in line number 87 please remove “were” from sentence.*

Done

*4-In line number 124 please add comma after “(Leica S6D)”.*

Done

*5-in line number 138 please add “was” before “done”.*

Done

*Results:*

*1-In line number 167 please change “on those were presented in Table 2” to “on those eggs are presented in Table 2.*

Done

*2-In line number 180 please change “temperature were given in Table 3” to “temperature are given in Table 3.”*

Done

*3-In line number 192 please change “days was shown in Table 4.” to “days is shown in Table 4.”*

Done

*4-In line number 202 please change “eggs were presented in Table 5” to “eggs are presented in Table 5.”*

Done

*Discussion: Well written and explained*

*Conclusion:*

*1-In line number please remove “at”.*

Done

*2-in line number 330 please add “for” before up to.*

Done

*References:*

*This sections needs some formatting*

*Done*

To reviewer 2:

Your general critics towards to the manuscript have been completely double checked by all the coauthors and revised also according to other reviewers’ suggestions. Your published papers that you suggested me to cite is irrelevant to the topic of the manuscript. So, I’m sorry not to cite your papers in the manuscript due to ethical considerations.

To reviewer 3:

I’m grateful for you to spend your time to improve the quality of the manuscript. Here I have the answers of your suggestions below:

*“The strong claim that storage from 3 to 21°C has "no serious negative effects" should be moderated because the results show specific significant negative effects (e.g., lower chick weight and longer incubation at 15°C, increased middle stage mortality at 21°C for 7 days). The abstract and conclusion should reflect these nuances.”*

Done.

*“Line 19: "The study was designed to 2 storage durations" → Should be "The study was designed as a 2 × 4 factorial arrangement with 2 storage durations"*

Done.

*“Line 20: "a total of 480 eggs (60 eggs per each subgroup) was directly sent" → "were sent" (subject-verb agreement)”*

Done

Line 24: "longer incubation rate" → Unclear phrasing. Should be "longer incubation duration" or "extended incubation period"

Done.

Line 25: "Almost all egg and hatching traits were found to be similar" → This overgeneralizes. Results show significant differences in specific traits (weight loss, chick weight, incubation length).

That is why the sentences starts with “almost”: not to overgeneralize the whole results.

*“Line 31-34: Strong claim about "no serious negative effect" needs qualification given the significant effects observed at 15°C and 21°C.”*

Even though there are few parameters that had significant effect on storage temperature, it did not affect the general results like hatchability and embryonic development (EGK>10) of which are the most important parameters in poultry hatching. So, anyone can conclude from the study that there was no significant differences between treatment groups affecting the general hatching performance. Also, according to the guideleness of the journal, the abstract should be written in brief and simple language just to show the general outcomes.

*“Line 35-37: "would storage" → Grammatical error, should be "could store"”*

Done

*“Line 38-42: Conclusion seems stronger than what the data supports. Consider tempering language.”*

Some revisions in conclusion were done according to your suggestions.

*“In Table 2, the superscripts for significant differences in EWLS are missing. The authors should add them.”*

Checked

The physiological zero for chukar partridge may be below 21°C, which is a finding of the study. The introduction should not state it as 21°C as a fact for partridges, but rather as a general concept or for other species.

The discussion should more directly address the counter-intuitive results at 15°C and the implications of the embryonic development at 21°C.

Done
