# Supplementary material for: Large variability of preincubation storage temperature in chukar partridge eggs under normal storage duration: Impact on egg and hatching traits, and embryonic development
Source: PLoS One. 2026 Sep 23;21(9):e0354754. doi: 10.1371/journal.pone.0354754 (PMC13600485; doi:10.1371/journal.pone.0354754)
Supplement: Supplementary file 4 [file pone.0354754.s004.docx]

To reviewer 4:

Dear Reviewer;

The embryonic developmental stages were determined according to the Eyal-Giladi and Kochav (EGK) staging system, which was developed as a complementary normal table for the pre-primitive-streak period of avian development (Eyal-Giladi and Kochav, 1976). EGK subdivides the interval from fertilization to just before primitive streak formation into 14 stages (I–XIV), and in avian embryology it is commonly used together with the Hamburger–Hamilton system for later developmental stages (Hamburger and Hamilton, 1951; Stern, 2018). Morphological staging was preferred over incubation time because embryos incubated for the same duration may differ considerably in developmental progression, whereas stage tables provide a more precise and reproducible basis for comparison (Stern, 2018). Comparative studies have further shown that, although the rate of development differs among avian species, embryos assigned to the same morphological stage exhibit nearly identical external features (Sellier et al., 2006). Therefore, the use of the EGK system as a comparative avian framework for staging partridge embryos is methodologically justified.

*Eyal-Giladi, H., & Kochav, S. (1976). From cleavage to primitive streak formation: a complementary normal table and a new look at the first stages of the development of the chick: I. General morphology. Developmental biology, 49(2), 321-337.*

*Hamburger, V., & Hamilton, H. L. (1992). A series of normal stages in the development of the chick embryo. Developmental dynamics, 195(4), 231-272.*

*Stern, C. D. (2018). Staging tables for avian embryos: a little history. International Journal of Developmental Biology, 62(1-3), 43-48.*

*Sellier, N., Brillard, J. P., Dupuy, V., & Bakst, M. R. (2006). Comparative staging of embryo development in chicken, turkey, duck, goose, guinea fowl, and Japanese quail assessed from five hours after fertilization through seventy-two hours of incubation. Journal of Applied Poultry Research, 15(2), 219-228.*

That your suggestion about chemical composition is probably misunderstanding. The title of the manuscript includes hatching performance and embryonic development. We did not investigate chemical composition which was out of our hypothesis in this study.

We discussed our results with all the relevant articles up to date. Due to originality of the topic and very limited research on storage temperature in partridge eggs, we cannot cite more research. In addition, I would like to point out we already have 29 citations in the manuscript.
