# Supplementary material for: Large variability of preincubation storage temperature in chukar partridge eggs under normal storage duration: Impact on egg and hatching traits, and embryonic development
Source: PLoS One. 2026 Sep 23;21(9):e0354754. doi: 10.1371/journal.pone.0354754 (PMC13600485; doi:10.1371/journal.pone.0354754)
Supplement: Peer review history

# Peer review: Decision Letter 0

**DOI:** 10.1371/journal.pone.0354754.r001

**Authors:** Fazul Nabi

12 Nov 2025

PONE-D-25-50845Large variability of preincubation storage temperature in chukar partridge eggs under normal storage duration: Impact on egg and hatching traits, and embryonic developmentPLOS ONE

Dear Dr.

Thank you for submitting your manuscript to PLOS ONE. After careful consideration, we feel that it has merit but does not fully meet PLOS ONE’s publication criteria as it currently stands. Therefore, we invite you to submit a revised version of the manuscript that addresses the points raised during the review process.

Please submit your revised manuscript by Dec 27 2025 11:59PM. If you will need more time than this to complete your revisions, please reply to this message or contact the journal office at plosone@plos.org. When you're ready to submit your revision, log on to https://www.editorialmanager.com/pone/ and select the 'Submissions Needing Revision' folder to locate your manuscript file.

Please include the following items when submitting your revised manuscript:

- A rebuttal letter that responds to each point raised by the academic editor and reviewer(s). You should upload this letter as a separate file labeled 'Response to Reviewers'.
- A marked-up copy of your manuscript that highlights changes made to the original version. You should upload this as a separate file labeled 'Revised Manuscript with Track Changes'.
- An unmarked version of your revised paper without tracked changes. You should upload this as a separate file labeled 'Manuscript'.

If you would like to make changes to your financial disclosure, please include your updated statement in your cover letter. Guidelines for resubmitting your figure files are available below the reviewer comments at the end of this letter.

If applicable, we recommend that you deposit your laboratory protocols in protocols.io to enhance the reproducibility of your results. Protocols.io assigns your protocol its own identifier (DOI) so that it can be cited independently in the future. For instructions see: https://journals.plos.org/plosone/s/submission-guidelines#loc-laboratory-protocols. Additionally, PLOS ONE offers an option for publishing peer-reviewed Lab Protocol articles, which describe protocols hosted on protocols.io. Read more information on sharing protocols at https://plos.org/protocols?utm_medium=editorial-email&utm_source=authorletters&utm_campaign=protocols.

We look forward to receiving your revised manuscript.

Kind regards,

Fazul Nabi

Academic Editor

PLOS ONE

Journal Requirements:

When submitting your revision, we need you to address these additional requirements.

1. Please ensure that your manuscript meets PLOS ONE's style requirements, including those for file naming. The PLOS ONE style templates can be found at

https://journals.plos.org/plosone/s/file?id=wjVg/PLOSOne_formatting_sample_main_body.pdf and

https://journals.plos.org/plosone/s/file?id=ba62/PLOSOne_formatting_sample_title_authors_affiliations.pdf.

2. Thank you for stating the following financial disclosure:

“The study was funded by the Scientific Research Projects Coordination Unit, Selcuk University (Project Number: 22401120).”

Please state what role the funders took in the study.  If the funders had no role, please state: "The funders had no role in study design, data collection and analysis, decision to publish, or preparation of the manuscript."

If this statement is not correct you must amend it as needed.

Please include this amended Role of Funder statement in your cover letter; we will change the online submission form on your behalf.

3. In the online submission form you indicate that your data is not available for proprietary reasons and have provided a contact point for accessing this data. Please note that your current contact point is a co-author on this manuscript. According to our Data Policy, the contact point must not be an author on the manuscript and must be an institutional contact, ideally not an individual. Please revise your data statement to a non-author institutional point of contact, such as a data access or ethics committee, and send this to us via return email. Please also include contact information for the third party organization, and please include the full citation of where the data can be found.

4. Please be informed that funding information should not appear in the Acknowledgments section or other areas of your manuscript. We will only publish funding information present in the Funding Statement section of the online submission form. Please remove any funding-related text from the manuscript.

5. If the reviewer comments include a recommendation to cite specific previously published works, please review and evaluate these publications to determine whether they are relevant and should be cited. There is no requirement to cite these works unless the editor has indicated otherwise.

[Note: HTML markup is below. Please do not edit.]

Reviewers' comments:

Reviewer's Responses to Questions

**Comments to the Author**

1. Is the manuscript technically sound, and do the data support the conclusions?

The manuscript must describe a technically sound piece of scientific research with data that supports the conclusions. Experiments must have been conducted rigorously, with appropriate controls, replication, and sample sizes. The conclusions must be drawn appropriately based on the data presented.

Reviewer #1: Yes

Reviewer #2: Partly

Reviewer #3: Yes

**********

2. Has the statistical analysis been performed appropriately and rigorously?

Reviewer #1: Yes

Reviewer #2: Yes

Reviewer #3: Yes

**********

3. Have the authors made all data underlying the findings in their manuscript fully available?

The PLOS Data policy requires authors to make all data underlying the findings described in their manuscript fully available without restriction, with rare exception (please refer to the Data Availability Statement in the manuscript PDF file). The data should be provided as part of the manuscript or its supporting information, or deposited to a public repository. For example, in addition to summary statistics, the data points behind means, medians and variance measures should be available. If there are restrictions on publicly sharing data—e.g. participant privacy or use of data from a third party—those must be specified.

Reviewer #1: Yes

Reviewer #2: Yes

Reviewer #3: Yes

**********

4. Is the manuscript presented in an intelligible fashion and written in standard English?

PLOS ONE does not copyedit accepted manuscripts, so the language in submitted articles must be clear, correct, and unambiguous. Any typographical or grammatical errors should be corrected at revision, so please note any specific errors here.

Reviewer #1: No

Reviewer #2: No

Reviewer #3: No

**********

5. Review Comments to the Author

Please use the space provided to explain your answers to the questions above. You may also include additional comments for the author, including concerns about dual publication, research ethics, or publication ethics. (Please upload your review as an attachment if it exceeds 20,000 characters)

Reviewer #1: Reviewer’s comments regarding the manuscript titled “Large variability of preincubation storage temperature in chukar partridge eggs under normal storage duration: Impact on egg and hatching traits, and embryonic development”

Title: The title in line numbers 1-4 seems too long. Please make it more precise & shorter.

Abstract:

1-In line number 32 please add “were” after “the eggs”.

2-In line number 35 please change “on” to “in”.

3-In line number 38 please change “at from” to “between”.

4-In line number 41 please change “farmers would storage” to farmers would store”.

Introduction:

1-In line number 49 please change “valuable” to “valuability”.

2-In line number 60 please change “42 d” to “42 days”.

3-Please give reference at the end of line number 67.

4-In line number 70 please change “under” to “for”.

Materials and Methods:

1-In line number 82 please add “the” before department.

2-In line number 86 please add “was” before “created”.

3-in line number 87 please remove “were” from sentence.

4-In line number 124 please add comma after “(Leica S6D)”.

5-in line number 138 please add “was” before “done”.

Results:

1-In line number 167 please change “on those were presented in Table 2” to “on those eggs are presented in Table 2.

2-In line number 180 please change “temperature were given in Table 3” to “temperature are given in Table 3.”

3-In line number 192 please change “days was shown in Table 4.” to “days is shown in Table 4.”

4-In line number 202 please change “eggs were presented in Table 5” to “eggs are presented in Table 5.”

Discussion: Well written and explained

Conclusion:

1-In line number please remove “at”.

2-in line number 330 please add “for” before up to.

References:

This sections needs some formating

Reviewer #2: The manuscript is very poor written, there are many flaws and mistakes the data is insufficient therefore I suggest a major revision, not acceptable in this condition.

Major Revision

Title: Large variability of preincubation storage temperature in chukar partridge eggs under normal storage duration: Impact on egg and hatching traits, and embryonic development

Abstract

Line (24-26) Improve the hypothesis and object clearly state them

Method should be clearly stated what are the number of replicates, also mentioned the experimental trail in details.

Results in abstract also need correction and improvement further

Add more related key words

Introduction

Revise the introduction

Give the brief background about the topic

Clearly mentioned the research gaps

Include the significance of your study

I suggest thoroughly improve the introduction portion, cite recent papers:

Hepatoprotective Effects of Olive Leaf Extract Against Carbon Tetrachloride-Induced Oxidative Stress: In vivo and In-silico Insights into the Nrf2-NFκB Pathway. Journal of Molecular Histology, 56(1), 1-15. https://doi.org/10.1007/s10735-024-10325-y. and Bovine Lactoferrin alleviates aflatoxin B1 induced hepatic and renal injury in broilers by mediating Nrf2 signaling pathway. Poultry Science, 104316. https://doi.org/10.1016/j.psj.2024.104316

Materials and Methods

Provide a schematic diagram to elaborate the methodology used in this study

Material and methods section lack the references, please cite proper references in this section

Provide supplementary file about the whole process of data mining form online sources, with their links also.

Here are some papers that my help you, if they are relevant cite them in your paper.

1. https://doi.org/10.1007/s10735-024-10325-y

2. https://doi.org/10.1007/s00360-024-01578-w

3. https://doi.org/10.1016/j.psj.2024.104316

4. https://doi.org/10.1080/00439339.2025.2522902

5. https://doi.org/10.1080/00439339.2025.2519329

6. https://doi.org/10.1080/00439339.2025.2468696

7. https://doi.org/10.5851/kosfa.2024.e130

Results and discussion

The results are not properly elaborate, improve this section thoroughly

The quality of tables is very poor I suggest to improve them according to the journal style. Also add some figures to the paper that are easy to read rather than tables.

The quality of paper increase if you can add histological studies and genetic level study in your manuscript.

Reviewer #3: This is a well-structured manuscript presenting a valuable study on chukar partridge egg storage. The experimental design is sound, and the findings have practical significance for game bird breeders.

Here are technical comments:

The strong claim that storage from 3 to 21°C has "no serious negative effects" should be moderated because the results show specific significant negative effects (e.g., lower chick weight and longer incubation at 15°C, increased middle stage mortality at 21°C for 7 days). The abstract and conclusion should reflect these nuances.

Line 19: "The study was designed to 2 storage durations" → Should be "The study was designed as a 2 × 4 factorial arrangement with 2 storage durations"

Line 20: "a total of 480 eggs (60 eggs per each subgroup) was directly sent" → "were sent" (subject-verb agreement)

Line 24: "longer incubation rate" → Unclear phrasing. Should be "longer incubation duration" or "extended incubation period"

Line 25: "Almost all egg and hatching traits were found to be similar" → This overgeneralizes. Results show significant differences in specific traits (weight loss, chick weight, incubation length).

Line 31-34: Strong claim about "no serious negative effect" needs qualification given the significant effects observed at 15°C and 21°C.

Line 35-37: "would storage" → Grammatical error, should be "could store"

Line 38-42: Conclusion seems stronger than what the data supports. Consider tempering language.

In Table 2, the superscripts for significant differences in EWLS are missing. The authors should add them.

The physiological zero for chukar partridge may be below 21°C, which is a finding of the study. The introduction should not state it as 21°C as a fact for partridges, but rather as a general concept or for other species.

The discussion should more directly address the counter-intuitive results at 15°C and the implications of the embryonic development at 21°C.

**********

6. PLOS authors have the option to publish the peer review history of their article (what does this mean?). If published, this will include your full peer review and any attached files.

If you choose “no”, your identity will remain anonymous but your review may still be made public.

**Do you want your identity to be public for this peer review?**  For information about this choice, including consent withdrawal, please see our Privacy Policy.

Reviewer #1: **Yes:**  Syed Zahid Ali Shah

Reviewer #2: No

Reviewer #3: **Yes:**  Hafiz Ishfaq Ahmad

**********

[NOTE: If reviewer comments were submitted as an attachment file, they will be attached to this email and accessible via the submission site. Please log into your account, locate the manuscript record, and check for the action link "View Attachments". If this link does not appear, there are no attachment files.]

To ensure your figures meet our technical requirements, please review our figure guidelines: https://journals.plos.org/plosone/s/figures

You may also use PLOS’s free figure tool, NAAS, to help you prepare publication quality figures: https://journals.plos.org/plosone/s/figures#loc-tools-for-figure-preparation.

NAAS will assess whether your figures meet our technical requirements by comparing each figure against our figure specifications.

Attachment Submitted filename: Revised manuscript clean.docx

# Author comment: Author response to Decision Letter 1

**DOI:** 10.1371/journal.pone.0354754.r002

18 Nov 2025

I'd like you to explain that the 2nd reviewer had general critics for the quality of the paper and gave no details, showing no guidence and leaving us revise the manuscript meaningfully. I also realized from his comments that the reviewer did not read the manuscript due to his suggestion about adding several citations to the manuscript of which were completely irrelevant. These citations would probably be from his papers because all of them were written the similar authors and completely irrelevant to the topic of our paper. Due to this kind of ethical consideration: I was unable to revise the paper according to his suggestions. Even though his bad comments and major revision decision: the other 2 reviewers had quite meaningful and fair comments that I already revised the manuscript in the light of their comments.

Attachment Submitted filename: Response to reviewers.docx

# Peer review: Decision Letter 1

**DOI:** 10.1371/journal.pone.0354754.r003

**Authors:** Wenpeng You

13 Apr 2026

PONE-D-25-50845R1Large variability of preincubation storage temperature in chukar partridge eggs under normal storage duration: Impact on egg and hatching traits, and embryonic developmentPLOS One

Dear Dr. Çam,

Thank you for submitting your manuscript to PLOS ONE. After careful consideration, we feel that it has merit but does not fully meet PLOS ONE’s publication criteria as it currently stands. Therefore, we invite you to submit a revised version of the manuscript that addresses the points raised during the review process.<section class="text-token-text-primary w-full focus:outline-none [--shadow-height:45px] has-data-writing-block:pointer-events-none has-data-writing-block:-mt-(--shadow-height) has-data-writing-block:pt-(--shadow-height) [&:has([data-writing-block])>*]:pointer-events-auto scroll-mt-[calc(var(--header-height)+min(200px,max(70px,20svh)))]" data-scroll-anchor="true" data-testid="conversation-turn-4" data-turn="assistant" data-turn-id="request-WEB:f3954855-84ce-4a0e-896c-884a39426cfe-1" dir="auto">

The manuscript shows merit but requires **major revision**  before it can be considered for publication. Key concerns remain regarding the depth of discussion, use of appropriate species-specific references, and justification of methodological decisions, particularly the classification of embryonic mortality stages . Please address these issues thoroughly in your revision.

Please submit your revised manuscript by May 28 2026 11:59PM. If you will need more time than this to complete your revisions, please reply to this message or contact the journal office at plosone@plos.org. When you're ready to submit your revision, log on to https://www.editorialmanager.com/pone/ and select the 'Submissions Needing Revision' folder to locate your manuscript file.

Please include the following items when submitting your revised manuscript:

</section>

- A letter that responds to each point raised by the academic editor and reviewer(s). You should upload this letter as a separate file labeled 'Response to Reviewers'.
- A marked-up copy of your manuscript that highlights changes made to the original version. You should upload this as a separate file labeled 'Revised Manuscript with Track Changes'.
- An unmarked version of your revised paper without tracked changes. You should upload this as a separate file labeled 'Manuscript'.

If you would like to make changes to your financial disclosure, please include your updated statement in your cover letter. Guidelines for resubmitting your figure files are available below the reviewer comments at the end of this letter.

If applicable, we recommend that you deposit your laboratory protocols in protocols.io to enhance the reproducibility of your results. Protocols.io assigns your protocol its own identifier (DOI) so that it can be cited independently in the future. For instructions see: https://journals.plos.org/plosone/s/submission-guidelines#loc-laboratory-protocols. Additionally, PLOS ONE offers an option for publishing peer-reviewed Lab Protocol articles, which describe protocols hosted on protocols.io. Read more information on sharing protocols at https://plos.org/protocols?utm_medium=editorial-email&utm_source=authorletters&utm_campaign=protocols.

As the corresponding author, your ORCID iD is verified in the submission system and will appear in the published article. PLOS supports the use of ORCID, and we encourage all coauthors to register for an ORCID iD and use it as well. Please encourage your coauthors to verify their ORCID iD within the submission system before final acceptance, as unverified ORCID iDs will not appear in the published article. *Only*  the individual author can complete the verification step; PLOS staff *cannot*  verify ORCID iDs on behalf of authors.

We look forward to receiving your revised manuscript.

Kind regards,

Wenpeng You

Academic Editor

PLOS One

Journal Requirements:

If the reviewer comments include a recommendation to cite specific previously published works, please review and evaluate these publications to determine whether they are relevant and should be cited. There is no requirement to cite these works unless the editor has indicated otherwise.

Additional Editor Comments:

<section class="text-token-text-primary w-full focus:outline-none [--shadow-height:45px] has-data-writing-block:pointer-events-none has-data-writing-block:-mt-(--shadow-height) has-data-writing-block:pt-(--shadow-height) [&:has([data-writing-block])>*]:pointer-events-auto scroll-mt-[calc(var(--header-height)+min(200px,max(70px,20svh)))]" data-scroll-anchor="true" data-testid="conversation-turn-4" data-turn="assistant" data-turn-id="request-WEB:f3954855-84ce-4a0e-896c-884a39426cfe-1" dir="auto">

The manuscript shows merit but requires **major revision**  before it can be considered for publication. Key concerns remain regarding the depth of discussion, use of appropriate species-specific references, and justification of methodological decisions, particularly the classification of embryonic mortality stages . Please address these issues thoroughly in your revision.

[Note: HTML markup is below. Please do not edit.]

Reviewers' comments:

Reviewer's Responses to Questions</section>

**Comments to the Author**

1. If the authors have adequately addressed your comments raised in a previous round of review and you feel that this manuscript is now acceptable for publication, you may indicate that here to bypass the “Comments to the Author” section, enter your conflict of interest statement in the “Confidential to Editor” section, and submit your "Accept" recommendation.

Reviewer #1: All comments have been addressed

Reviewer #3: All comments have been addressed

Reviewer #4: All comments have been addressed

Reviewer #5: (No Response)

**********

2. Is the manuscript technically sound, and do the data support the conclusions?

The manuscript must describe a technically sound piece of scientific research with data that supports the conclusions. Experiments must have been conducted rigorously, with appropriate controls, replication, and sample sizes. The conclusions must be drawn appropriately based on the data presented.

Reviewer #1: Yes

Reviewer #3: Yes

Reviewer #4: Partly

Reviewer #5: Yes

**********

3. Has the statistical analysis been performed appropriately and rigorously?

Reviewer #1: Yes

Reviewer #3: Yes

Reviewer #4: Yes

Reviewer #5: Yes

**********

4. Have the authors made all data underlying the findings in their manuscript fully available?

The PLOS Data policy requires authors to make all data underlying the findings described in their manuscript fully available without restriction, with rare exception (please refer to the Data Availability Statement in the manuscript PDF file). The data should be provided as part of the manuscript or its supporting information, or deposited to a public repository. For example, in addition to summary statistics, the data points behind means, medians and variance measures should be available. If there are restrictions on publicly sharing data—e.g. participant privacy or use of data from a third party—those must be specified.

Reviewer #1: Yes

Reviewer #3: Yes

Reviewer #4: Yes

Reviewer #5: Yes

**********

5. Is the manuscript presented in an intelligible fashion and written in standard English?

PLOS ONE does not copyedit accepted manuscripts, so the language in submitted articles must be clear, correct, and unambiguous. Any typographical or grammatical errors should be corrected at revision, so please note any specific errors here.

Reviewer #1: Yes

Reviewer #3: Yes

Reviewer #4: Yes

Reviewer #5: Yes

**********

6. Review Comments to the Author

Please use the space provided to explain your answers to the questions above. You may also include additional comments for the author, including concerns about dual publication, research ethics, or publication ethics. (Please upload your review as an attachment if it exceeds 20,000 characters)

Reviewer #1: (No Response)

Reviewer #3: All comments have been addressed carefully and technically and the manuscript is now suitable for publication.

Reviewer #4: - in this study, the Authors did not depended on referenced that special in same species of bird and he depended on references that talking with chickens and I see that is wrong because it have a different development and hatching time.

- you must search the differences in chemical composition of yolk and albumen because in storing time it change their PH because of losing water and co2.

-your discussion is very poor and depended on few references just.

-in embryo mortality, you cant just depending on broiler embryos references because its different in embryo stages and hatching time.

- you separated the embryonic mortalities for 3 stages?? what is the scientific base that you depend on ?

Reviewer #5: The manuscript presents a study on a missing part of temperature variation on storage. "Large variability of preincubation storage temperature in chukar partridge eggs under normal storage duration: Impact on egg and hatching traits, and embryonic development" is filling those points in literature. The experimental design is appropriate, and the manuscript is written clearly. The conclusions are well-supported by the data presented. I have no further recommendations for improvement and suggest acceptance in its current form.

**********

7. PLOS authors have the option to publish the peer review history of their article (what does this mean?). If published, this will include your full peer review and any attached files.

If you choose “no”, your identity will remain anonymous but your review may still be made public.

**Do you want your identity to be public for this peer review?**  For information about this choice, including consent withdrawal, please see our Privacy Policy.

Reviewer #1: **Yes:**  Prof. Dr. Syed Zahid Ali Shah

Reviewer #3: No

Reviewer #4: **Yes:**  Prof. DR. Mohammed A. AL-Bayar

Reviewer #5: **Yes:**  Emre Sunay Gebes

**********

[NOTE: If reviewer comments were submitted as an attachment file, they will be attached to this email and accessible via the submission site. Please log into your account, locate the manuscript record, and check for the action link "View Attachments". If this link does not appear, there are no attachment files.]

To ensure your figures meet our technical requirements, please review our figure guidelines: https://journals.plos.org/plosone/s/figures

You may also use PLOS’s free figure tool, NAAS, to help you prepare publication quality figures: https://journals.plos.org/plosone/s/figures#loc-tools-for-figure-preparation.

NAAS will assess whether your figures meet our technical requirements by comparing each figure against our figure specifications.

Attachment Submitted filename: PONE-D-25-50845_R1.pdf

# Author comment: Author response to Decision Letter 2

**DOI:** 10.1371/journal.pone.0354754.r004

28 May 2026

Dear Reviewer;

The embryonic developmental stages were determined according to the Eyal-Giladi and Kochav (EGK) staging system, which was developed as a complementary normal table for the pre-primitive-streak period of avian development (Eyal-Giladi and Kochav, 1976). EGK subdivides the interval from fertilization to just before primitive streak formation into 14 stages (I–XIV), and in avian embryology it is commonly used together with the Hamburger–Hamilton system for later developmental stages (Hamburger and Hamilton, 1951; Stern, 2018). Morphological staging was preferred over incubation time because embryos incubated for the same duration may differ considerably in developmental progression, whereas stage tables provide a more precise and reproducible basis for comparison (Stern, 2018). Comparative studies have further shown that, although the rate of development differs among avian species, embryos assigned to the same morphological stage exhibit nearly identical external features (Sellier et al., 2006). Therefore, the use of the EGK system as a comparative avian framework for staging partridge embryos is methodologically justified.

Eyal-Giladi, H., & Kochav, S. (1976). From cleavage to primitive streak formation: a complementary normal table and a new look at the first stages of the development of the chick: I. General morphology. Developmental biology, 49(2), 321-337.

Hamburger, V., & Hamilton, H. L. (1992). A series of normal stages in the development of the chick embryo. Developmental dynamics, 195(4), 231-272.

Stern, C. D. (2018). Staging tables for avian embryos: a little history. International Journal of Developmental Biology, 62(1-3), 43-48.

Sellier, N., Brillard, J. P., Dupuy, V., & Bakst, M. R. (2006). Comparative staging of embryo development in chicken, turkey, duck, goose, guinea fowl, and Japanese quail assessed from five hours after fertilization through seventy-two hours of incubation. Journal of Applied Poultry Research, 15(2), 219-228.

That your suggestion about chemical composition is probably misunderstanding. The title of the manuscript includes hatching performance and embryonic development. We did not investigate chemical composition which was out of our hypothesis in this study.

We discussed our results with all the relevant articles up to date. Due to originality of the topic and very limited research on storage temperature in partridge eggs, we cannot cite more research. In addition, I would like to point out we already have 29 citations in the manuscript.

Attachment Submitted filename: Response to reviewer.docx

# Peer review: Decision Letter 2

**DOI:** 10.1371/journal.pone.0354754.r005

**Authors:** Wenpeng You

12 Jul 2026

Large variability of preincubation storage temperature in chukar partridge eggs under normal storage duration: Impact on egg and hatching traits, and embryonic development

PONE-D-25-50845R2

Dear Dr. Çam,

We’re pleased to inform you that your manuscript has been judged scientifically suitable for publication and will be formally accepted for publication once it meets all outstanding technical requirements.

Within one week, you’ll receive an e-mail detailing the required amendments. When these have been addressed, you’ll receive a formal acceptance letter and your manuscript will be scheduled for publication.

An invoice will be generated when your article is formally accepted. Please note, if your institution has a publishing partnership with PLOS and your article meets the relevant criteria, all or part of your publication costs will be covered. Please make sure your user information is up-to-date by logging into Editorial Manager at Editorial Manager® and clicking the ‘Update My Information' link at the top of the page. For questions related to billing, please contact billing support.

If your institution or institutions have a press office, please notify them about your upcoming paper to help maximize its impact. If they’ll be preparing press materials, please inform our press team as soon as possible -- no later than 48 hours after receiving the formal acceptance. Your manuscript will remain under strict press embargo until 2 pm Eastern Time on the date of publication. For more information, please contact onepress@plos.org.

Kind regards,

Wenpeng You

Academic Editor

PLOS One

Additional Editor Comments (optional):

Congratulations!

Reviewers' comments:

Reviewer's Responses to Questions

**Comments to the Author**

1. If the authors have adequately addressed your comments raised in a previous round of review and you feel that this manuscript is now acceptable for publication, you may indicate that here to bypass the “Comments to the Author” section, enter your conflict of interest statement in the “Confidential to Editor” section, and submit your "Accept" recommendation.

Reviewer #1: All comments have been addressed

Reviewer #5: All comments have been addressed

**********

2. Is the manuscript technically sound, and do the data support the conclusions?

The manuscript must describe a technically sound piece of scientific research with data that supports the conclusions. Experiments must have been conducted rigorously, with appropriate controls, replication, and sample sizes. The conclusions must be drawn appropriately based on the data presented.

Reviewer #1: Yes

Reviewer #5: Yes

**********

3. Has the statistical analysis been performed appropriately and rigorously?

Reviewer #1: I Don't Know

Reviewer #5: Yes

**********

4. Have the authors made all data underlying the findings in their manuscript fully available?

The PLOS Data policy requires authors to make all data underlying the findings described in their manuscript fully available without restriction, with rare exception (please refer to the Data Availability Statement in the manuscript PDF file). The data should be provided as part of the manuscript or its supporting information, or deposited to a public repository. For example, in addition to summary statistics, the data points behind means, medians and variance measures should be available. If there are restrictions on publicly sharing data—e.g. participant privacy or use of data from a third party—those must be specified.

Reviewer #1: Yes

Reviewer #5: Yes

**********

5. Is the manuscript presented in an intelligible fashion and written in standard English?

PLOS ONE does not copyedit accepted manuscripts, so the language in submitted articles must be clear, correct, and unambiguous. Any typographical or grammatical errors should be corrected at revision, so please note any specific errors here.

Reviewer #1: Yes

Reviewer #5: Yes

**********

6. Review Comments to the Author

Please use the space provided to explain your answers to the questions above. You may also include additional comments for the author, including concerns about dual publication, research ethics, or publication ethics. (Please upload your review as an attachment if it exceeds 20,000 characters)

Reviewer #1: The authors have already did the changes and the manuscript is much better than the originally submitted article.

Reviewer #5: The authors have satisfactorily addressed all comments from the previous review rounds. The methodology is scientifically justified, the statistical approach is robust, and the paper provides valuable, practical insights for the poultry industry. I am completely satisfied with this revision and recommend the manuscript for immediate publication.

**********

7. PLOS authors have the option to publish the peer review history of their article (what does this mean?). If published, this will include your full peer review and any attached files.

If you choose “no”, your identity will remain anonymous but your review may still be made public.

**Do you want your identity to be public for this peer review?**  For information about this choice, including consent withdrawal, please see our Privacy Policy.

Reviewer #1: **Yes:**  Syed Zahid Ali Shah

Reviewer #5: **Yes:**  EMRE SUNAY GEBES

**********

# Editor report: Acceptance letter

**DOI:** 10.1371/journal.pone.0354754.r006

**Authors:** Wenpeng You

PONE-D-25-50845R2

PLOS One

Dear Dr. Çam,

I'm pleased to inform you that your manuscript has been deemed suitable for publication in PLOS One. Congratulations! Your manuscript is now being handed over to our production team.

At this stage, our production department will prepare your paper for publication. This includes ensuring the following:

* All references, tables, and figures are properly cited

* All relevant supporting information is included in the manuscript submission,

* There are no issues that prevent the paper from being properly typeset

You will receive further instructions from the production team, including instructions on how to review your proof when it is ready. Please keep in mind that we are working through a large volume of accepted articles, so please give us a few days to review your paper and let you know the next and final steps.

Lastly, if your institution or institutions have a press office, please let them know about your upcoming paper now to help maximize its impact. If they'll be preparing press materials, please inform our press team within the next 48 hours. Your manuscript will remain under strict press embargo until 2 pm Eastern Time on the date of publication. For more information, please contact onepress@plos.org.

You will receive an invoice from PLOS for your publication fee after your manuscript has reached the completed accept phase. If you receive an email requesting payment before acceptance or for any other service, this may be a phishing scheme. Learn how to identify phishing emails and protect your accounts at https://explore.plos.org/phishing.

If we can help with anything else, please email us at customercare@plos.org.

Thank you for submitting your work to PLOS ONE and supporting open access.

Kind regards,

PLOS ONE Editorial Office Staff

on behalf of

Dr. Wenpeng You

%CORR_ED_EDITOR_ROLE%

PLOS One
